# Type A and D *Clostridium perfringens* with unique genetic evolution characteristics cause mortality in juvenile Bactrian camels

**DOI:** 10.3389/fcimb.2026.1787432

**Published:** 2026-05-29

**Authors:** Wanpeng Ma, Yueqi Li, Huaibin Yao, Jie Ren, Junxiang Wei, Xueting Zhao, Lin Zhang, Zhanqiang Su

**Affiliations:** 1 College of Veterinary Medicine, Xinjiang Agricultural University, Urumqi, China; 2 Xinjiang Key Laboratory of New Drug Research and Development for Herbivorous AnimaIs, Urumqi, China; 3 Xinjiang Laboratory of Special Environmental Microbiology, Institute of Microbiology, Xinjiang Academy of Agricultural Sciences, Urumqi, China

**Keywords:** Bactrian camel, clostridium perfringens, juvenile camel, pathological changes, physiological indicators, whole-genome sequencing

## Abstract

**Research Background:**

*Clostridium perfringens* (*C. perfringens*) disease in Bactrian camels is an acute infectious disease with high mortality, causing serious economic losses to the breeding industry. However, studies on its epidemiological patterns and whole-genome characteristics remain limited.

**Objective:**

This study aimed to investigate the epidemiological patterns and whole-genome characteristics of *C. perfringens* disease in Bactrian camels.

**Methods:**

Epidemiological investigations, physiological parameter measurements, pathological analyses, isolation and identification of *C. perfringens*, and whole-genome sequencing were performed to elucidate the epidemiological patterns and whole-genome characteristics of *C. perfringens* disease in Bactrian camels.

**Results:**

The incidence of *C. perfringens* disease in Bactrian camels was 37.89% (72/190), with a mortality rate of 54.17% (39/72). Compared with healthy camels, affected camels exhibited elevated body temperature, respiration, and pulse, along with significantly increased hemoglobin levels and red cell distribution width (*p* < 0.01). Elevated alkaline phosphatase, amylase, and phosphorus levels were also observed (*P* < 0.01). Autopsy revealed hemorrhagic gastroenteritis; enlargement of the liver, kidneys, spleen, and other organs; and cerebral congestion and edema. Histopathological examination revealed edema, hemorrhage, and congestion in parenchymal organs, along with reduced neuronal numbers and neuronal shrinkage in brain tissue. Three toxin genes, *plc, etx* and *cpe*, were detected by polymerase chain reaction. Whole-genome sequencing analysis revealed that the total genome length of the eight strains of *C. perfringens* ranged from 3, 195, 592 bp to 3, 595, 833 bp, and the predicted number of coding genes ranged from 2, 897 to 3, 413. Gene Ontology database annotation indicated enrichment in metabolic processes, cellular processes, catalytic activity, binding, and cellular component categories. Kyoto Encyclopedia of Genes and Genomes annotation identified pathways related to cellular community−prokaryotes, signaling molecules and interactions, translation, and metabolism. Virulence Factors of Pathogenic Bacteria Database annotation identified virulence factors, including alpha-toxin, *C. perfringens* enterotoxin, theta-toxin, alpha-clostripain, kappa-toxin, mu-toxin, and sialidase. Six drug resistance genes (*vanh*, *cpir*, *vant*, *vanw*, *vany* and *mPRF*) were annotated by the Comprehensive Antibiotic Resistance Database. In addition, three novel sequence types (ST1057, ST1058, and ST1059) were identified. Phylogenetic analysis based on the core genome revealed that the strains isolated from Bactrian camel showed substantial genomic divergence relative to strains isolated from other animals.

**Conclusion:**

In young Bactrian camels, *C. perfringens* is predominantly associated with type A and D strains, which exhibit distinct genomic characteristics, providing a foundation for improved diagnosis, prevention, and vaccine development.

## 1 Introduction

Camels are relatively specialized herbivorous livestock that play an essential role in animal husbandry and agriculture in arid and semi-arid areas, providing important resources such as milk, meat, and hair for farmers and herders (Yao et al., 2023). Owing to their long-term growth in desert and semi-desert areas (Tao et al., 2023), camels are exposed to a variety of pathogens; however, their strong resilience often results in mild or atypical clinical manifestations (Fayez et al., 2013; Elhelw et al., 2022). Camels are reportedly more vulnerable to certain pathogens; for example, the incidence rate of *Clostridium perfringens* (*C. perfringens*) in camels is significantly higher than that in other livestock (Fayez et al., 2013). *C. perfringens* disease, also known as enterotoxemia (Hm et al., 2024), is an acute infectious zoonotic disease characterized by high morbidity and mortality (Hussain et al., 2022). Clinically, the disease is associated with abdominal pain, diarrhea, fever, local edema, and skeletal muscle necrosis, which can rapidly progress to toxemia, sepsis, shock, and death (Navarro et al., 2020).

*C. perfringens* is an anaerobic, non-motile, spore-forming Gram-positive bacterium with strong resistance to high-temperature, aerobic, and low-nutrient environments. Consequently, it is widely distributed in human and animal intestines, as well as soil and sewage, and represents a common foodborne pathogen (Li et al., 2016; Fayez et al., 2021). *C. perfringens* is associated with systemic and intestinal diseases in humans and animals, and its pathogenicity is primarily mediated by the production of multiple toxins and enzymes (Kiu and Hall, 2018), including alpha, beta, epsilon, and iota toxins; enterotoxins; and necrotic enterotoxins (Hm et al., 2024; Mubarak et al., 2024), which are encoded by genes such as *cpa/plc, cpb, etx, iap/ibp/itx, cpe*, and *netB* (Grenda et al., 2023). *C. perfringens* has traditionally been classified into five types based on the toxin profiles: Type A bacteria produce alpha toxins; type B bacteria produce alpha, beta, and epsilon toxins; type C bacteria produce alpha and beta toxins; type D bacteria produce alpha and epsilon toxins; and type E bacteria produce alpha and iota toxins (Omer et al., 2020). Type A *C. perfringens* can cause gas gangrene and food poisoning in humans and enteritis in livestock and wild animals. Types B and C *C. perfringens* can cause enterotoxemia and necrotic enteritis in lambs, calves, piglets, and poultry. Type D *C. perfringens* can cause dysentery and medullary kidney damage in lambs. Type E *C. perfringens* can cause enteritis in sheep, rabbits, cows, and other animals (Omer et al., 2020; Moustafa et al., 2022). More recently, two new genotypes: type F (alpha toxin and *C. perfringens* enterotoxin [*cpe*]) and type G (alpha toxin and necrotic enteritis B-like toxin), were identified (Santos et al., 2022). No single strain produces all known toxins (Kiu and Hall, 2018).

Research on *C. perfringens* disease in camels remains extremely limited, with studies confined to pathogen isolation, identification, and detection (Hm et al., 2024). In Saudi Arabia, the reported prevalence of *C. perfringens* in camels is 56.3% (Fayez et al., 2020), with toxin types A, B, C, and D detected in stool samples of young camels. In addition, toxin types A (64.3%), B (7.1%), C (21.5%), D (7.1%), and *Clostridioides difficile* (*C. difficile*) have been isolated from camel meat (Fayez et al., 2013). Studies have detected toxin genes (*cpa, cpb*, and *etx*) by polymerase chain reaction (PCR), and beta and beta toxins have been identified in the cultured supernatant (Fayez et al., 2021). *C. perfringens* alpha toxin and *cpe* toxins were detected in the organs from sudden-death camels (Mubarak et al., 2024). In Iran, investigations focused primarily on bacterial detection without in-depth research, and *C. difficile* was not detected in the camel meat samples (Mohammadpour et al., 2020). In Pakistan, types A and D were isolated from the intestinal tract of sudden-death camels, with type D associated with higher mortality. Multiple PCR detected *cpa* and *cpb2* genes (Hm et al., 2024). In Egypt, the isolation rate of *C. perfringens* in diseased camels in Dakhalia Province was 25%, while the isolation rate in healthy camels was 20%, mainly types A and D. Notably, the isolation rate of *C. perfringens* in camels is significantly higher than that reported in cattle and sheep (Wahdan and Elhaig, 2024). In Sharkia Governorate, the isolation rate of *C. perfringens* from diarrheal fecal samples of camels was 18%, and the isolation rate from meat products was 4%. The isolated strains were mainly type A, with low antibacterial activity against penicillin and cephalosporins, and 28% of the isolated strains demonstrated strong biofilms (Ahmed et al., 2022). In addition, *C. perfringens* has also been reported in Mongolia (Moebuu et al., 1966), the United Arab Emirates (Wernery et al., 1992), Kenya (Younan and Gluecks, 2007), India (Gupta et al., 2015), and other regions. However, most of these reports are relatively outdated, some as early as 1966, and are largely limited to clinical symptoms, necropsy findings, and pathological observations. Consequently, these studies are relatively independent, lack systematic research on the pathogenesis, and are predominantly focused on humped camels.

*C. perfringens* is an important pathogen that causes diseases in young animals. Traditional approaches for its detection include isolation, identification, and PCR-based methods (Moustafa et al., 2022). With the rapid advancement of whole-genome sequencing, it has increasingly been adopted for the diagnosis of pathogenic microorganisms, rapid characterization of virulence traits, surveillance of epidemics, and epidemiological tracking. Genomic information obtained from whole-genome sequencing has been extensively applied in clinical practice (Zhang et al., 2024a), substantially deepening our understanding of the genetic diversity and pathogenic mechanisms of *C. perfringens* (Zhu et al., 2025). Zeng et al. employed whole-genome sequencing to analyze the genomic homology and distribution of the *cpb2* gene in *C. perfringens* isolated from piglets and environmental sources, thereby elucidating the association between β2 toxin and piglet diarrhea (Zeng et al., 2021). Santos et al. characterized the *C. perfringens* isolated from dairy farms and surrounding environments and demonstrated considerable phylogenetic diversity, as well as the presence of plasmids capable of transferring virulence and antibiotic resistance determinants (Santos et al., 2022). Furthermore, analysis based on core genome multi-locus sequence typing (cgMLST) and single nucleotide polymorphisms during the goat reproductive period revealed potential regional or cross-regional transmission of *C. perfringens*, along with functional differences in protein-coding genes (Feng et al., 2024). Beyond these hosts, whole-genome sequencing technology has been widely applied to investigate *C. perfringens* in sheep (Wu et al., 2022), chickens (Heidarpanah et al., 2024), elk (Zhang et al., 2024a), and humans (Yan et al., 2025). However, whole-genome sequencing of *C. perfringens* in camels has not yet been reported. Xinjiang, the primary breeding region for Bactrian camels in China, has recently undergone a transition from traditional free-range grazing to captive feeding. As breeding scales have expanded, increasing numbers of neonatal camels have experienced sudden death and hemorrhagic gastroenteritis, with the underlying etiology remaining unknown. To address this gap, we systematically investigated *C. perfringens* disease in Bactrian camels by assessing the disease incidence, measuring physiological indicators, and conducting necropsy and pathological examinations, in combination with bacterial isolation, identification, and whole-genome sequencing. This study provides a theoretical foundation for the prevention and treatment of *C. perfringens* disease and offers valuable insights for future vaccine development for Bactrian camels.

## 2 Materials and methods

### 2.1 Experimental animals

Young Bactrian camels used in this study were sourced from a farm in Altay, Xinjiang. The Altay region is located in the northern Xinjiang Uygur Autonomous Region (X) and is characterized by a cold continental climate, with an average annual temperature of 0.7–4.9 °C, extreme temperatures ranging from −romi to 42.2 °C, annual precipitation of 131–223 mm, and an annual evaporation of 1, 367–2, 066 mm. Bactrian camels in this region are primarily managed under free-range grazing conditions from April to November, during which they feed mainly on plants belonging to the families *Compositae*, *Polygonaceae*, *Salicaceae*, *Rosaceae*, *Myrtaceae*, *Labiatae*, *Convolvulaceae*, and *Cruciferae*, as well as the genera *Tribulus* and *Cynomorium*. These plants comprise three phyla, 32 families, and 97 genera, including one fern species, one gymnosperm species, and 125 angiosperm species representing 30 families and 95 genera. Among them, 69 species have forage value, 49 have medicinal value, and 54 have ecological restoration value. From November to March each year, camels are kept in captivity and mainly fed alfalfa, wheat straw, and small amounts of cornmeal. Between February and April 2024, the farm maintained 650 adult female camels and 190 newborn camels. During this period, multiple cases of sudden death and hemorrhagic gastroenteritis were observed among young camels.

### 2.2 Determination of physiological indicators of diseased young camels

A total of 26 diseased young camels and 36 healthy young camels were selected for measurement of body temperature, respiratory rate, and pulse rate. Rectal temperature was measured using a mercury thermometer for 5 min. Respiratory rate and heart rate were determined by auscultation using a stethoscope over a 1-min period. Jugular venous blood samples were collected from nine diseased young camels and 61 healthy camels using 5-mL ethylenediaminetetraacetic acid (EDTA) anticoagulant tubes and ordinary blood collection tubes. Routine hematological parameters were analyzed using an Exigo animal blood cell analyzer (Exigo EOS), and serum biochemical indices were measured using a Seamat fully automatic multifunctional biochemical analyzer (SMT-120VP). Data analysis was performed using GraphPad Prism 9.3.0 (Martín-Barrasa et al., 2023).

### 2.3 Sample collection and histopathological observation

Necropsies were performed immediately after the death of each young camel to assess gross pathological changes. Tissue samples (0.5–1.0 cm) from the brain, heart, liver, spleen, lungs, kidneys, pre-shoulder lymph nodes, rumen, reticulum, small intestine, and bladder were collected and fixed in 4% paraformaldehyde tissue fixative (Guangzhou Yongjin Biotechnology Co., Ltd., Guangzhou, China). After trimming, dehydration, embedding, sectioning, staining, and sealing, panoramic slide scanners (3DHISTECH, Hungary; DESK/MIDI/250/1000) and CaseViewer 2.4 software were employed for observation (Mahmood et al., 2017; Hm et al., 2024).

### 2.4 Bacterial isolation, identification, and 16S rRNA gene sequence analysis

Liver and intestinal tissues from deceased young camels were collected and stored at 4°C for bacterial isolation. Liver tissue smears were subjected to Gram staining to assess the presence of bacteria (Fayez et al., 2020). Intestinal contents were collected using sterile cotton swabs and inoculated into 5 mL liquid thioglycolate broth (FTG, Qingdao Haibo Biotechnology Co., Ltd., Qingdao, China), followed by anaerobic incubation at 37°C for 15 h. Subsequently, 100 µL of enriched FTG culture was streaked onto the tryptose sulfite cycloserine (TSC) agar containing 0.5% D-cycloserine (Qingdao Haibo Biotechnology Co., Ltd., Qingdao, China) and incubated anaerobically at 37°C for 15 h. Presumptive colonies were identified by Gram staining. Milk fermentation, motility, nitrate reduction, and lactose fermentation tests (Qingdao Haibo Biotechnology Co., Ltd., Qingdao, China) were performed according to the inspection standards for *C. perfringens* (GB 4789.13-2012). Confirmed isolates were preserved in 50% glycerol at −tyc (Zafar Khan et al., 2022). Genomic DNA was extracted using a bacterial genomic DNA extraction kit (Thermo Fisher Scientific Technology Co., Ltd., Shanghai, China) and PCR-amplified using primers designed by Baums et al. (**Table 1**) (Baums et al., 2004; Omer et al., 2020). The *plc/cpa, cpb, etx, iap, cpe, netB*, and *cpb2* genes were amplified in a 50-μL reaction volume. The PCR reaction consisted of 12.5 μL of 2× Taq Master Mix (Beijing All Gold Biology, Beijing, China), 1 μL each of upstream and downstream primers (10 μM), 2 μL of DNA extract, and 50 μL of double-distilled water (ddH_2_O). Following amplification, the PCR products were analyzed using 2% agarose gel electrophoresis and sent to Sanger Bioengineering Co., Ltd. (Shanghai, China) for Sanger sequencing. The sequencing results were compared using the National Center for Biotechnology Information Basic Local Alignment Search Tool (NCBI BLAST).

**Table 1:** Polymerase chain reaction primer sequence and annealing temperature.

Gene	Primer sequence 5’ to 3’	Amplicon size (bp)	Annealing temp (°C)
*plc/cpa* (α-toxin)	CPA5L: AGTCTACGCTTGGGATGGAA CPA5R: TTTCCTGGGTTGTCCATTTC	900	55
*cpb* (β-toxin)	CPBL: TCCTTTCTTGAGGGAGGATAAA CPBR: TGAACCTCCTATTTTGTATCCCA	612	55
*etx* (ϵ-toxin)	CPETXL: GGGGAACCCTCAGTAGTTTCA CPETXR: CCAGCTGGATTTGAGTTTAATG	396	55
*iap* (ι-toxin)	CPIL: AAACGCATTAAAGCTCACACC CPIR: CTGCATACCCTGGAATGGCT	293	55
*cpe* (Clostridium perfringens enterotoxin)	CPEF: GGAGATGGTTGGATATTAGG CPER: GGACCAGCAGTTGTAGATA	233	56
*cpb2* (β2-toxin)	CPBL: CAAGCAATTGGGGGAGTTTA CPBR: GCAGAATCAGGATTTTGACCA	200	55
*netB* (necrotic enteritis B-like toxin)	AKP78: GCTGGTGCTGGAATAAATGC AKP79: TCGCCATTGAGTAGTTTCCC	560	55-58

### 2.5 Bacterial genome analysis

#### 2.5.1 Whole-genome sequencing of bacteria

Genomic DNA was extracted from eight isolated strains using the STE method. The specific steps were as follows: The bacterial suspension was transferred to a 2 mL Eppendorf tube and centrifuged twice to collect the precipitate. Ten milligrams of the precipitate were mixed thoroughly with 600 μL of STE. Then, 60 μL of lysozyme was added, and the mixture was incubated in a 37 °C water bath for 30 min. After incubation, the sample was mixed, and 100 μL of 10% sodium dodecyl sulfate and 10 μL of proteinase K were added. The sample was incubated in a 65 °C water bath for 30 min, followed by centrifugation at 12, 500 rpm for 7 min. The supernatant was transferred to a new tube, mixed with an equal volume of phenol:chloroform:isoamyl alcohol (25:24:1) (Beijing Dingguo Changsheng Biotechnology Co., Ltd., Beijing, China) and centrifuged at 12, 500 rpm for 7 min. Then, three-quarters of the supernatant volume of pre-chilled isopropanol was added, and the mixture was incubated at −tcuba for 30 min to precipitate the DNA, followed by centrifugation at 12, 000 rpm for 10 min. The precipitate was washed twice with 75% ethanol and air-dried in a fume hood for 3–5 min. Subsequently, 50–100 μL of elution buffer and 2 μL of RNase were added to dissolve the precipitate, and the sample was incubated at 37 °C for 25 min to digest RNA. Finally, DNA integrity and purity were assessed by agarose gel electrophoresis and quantified using a Qubit^®^ 2.0 fluorometer (Thermo Scientific). Large DNA fragments were recovered using the fully automated BluePippin nucleic acid fragment recovery system, followed by end repair. Barcoding of samples was performed using the EXP-NBD104 kit (Oxford Nanopore Technologies, Oxford, UK) via a PCR-based approach. Fragment sizes were assessed using a fully automated AATI capillary electrophoresis system, and samples were pooled in equimolar amounts. The SQK-LSK109 ligation kit (Oxford Nanopore Technologies, Oxford, UK) was then used to construct a 1D Nanopore library. For Illumina sequencing, libraries were prepared using the NEBNext^®^ Ultra™ DNA Library Prep Kit (NEB, USA) through a series of steps, including end repair, addition of an A tail, addition of a sequencing adapter, purification, and PCR amplification. After the completion of the library construction, one strain each of type A (JLFS-2) and type D (JLFS-7) were selected for sequencing by Beijing Novogene Technology Co., Ltd. using the Nanopore PromethION and Illumina NovaSeq PE150 platforms. This was performed to obtain complete genome information for subsequent vaccine development. Raw Nanopore sequencing data were statistically analyzed using NanoPlot (version 1.29.1; https://github.com/wdecoster/NanoPlot), and genome assembly was performed using Unicycler (version 0.4.8; https://github.com/rrwick/Unicycler). Genomic assembly was performed using second-generation and third-generation data, and chromosome and plasmid sequences were screened (Zhang et al., 2024). The remaining 6 strains (JLFS-1, JLFS-3, JLFS-4, JLFS-5, JLFS-6, and JLFS-8) were sequenced using Illumina platform PE150 (Beijing Novogene Technology Co., Ltd., Beijing, China) to elucidate the genomic information of the isolated strains. Raw sequencing data were filtered using fastp (using --in1 input_1.fq.gz, --out1 out_1.fq.gz, --in2 input_2.fq.gz, --out2 out_2.fq.gz, -g, -q 5, -u 50, -n 15, -l 150, --min_trim_length 10, --overlap_diff_limit 1, --overlap_diff_percent_limit 10, -j output.json, -h output.html) to obtain clean reads. A *K-mer*−-mern statistical method was subsequently used to estimate the genome size. Specifically, assembly was performed using SOAPdenovo2 with different *K−mer* values (default: 95, 107, 119), and the assembly result with the fewest scaffolds was selected. The optimal *K-mer* was used, and other parameters (-d -u -R -F, etc.) were adjusted to obtain preliminary assembly results. Assembly was performed using SPAdes (v4.2.0) with different *K-mer* values (default: 99 and 127); the optimal *K-mer* was selected based on the project type, and the assembly result with the fewest scaffolds was chosen. Assembly was performed using ABySS (v2.1.5) with a *K-mer* 64. The resulting assembly was then combined with the assembly outputs from the other two software tools using CISA, and the assembly with the fewest scaffolds was selected. Gaps in the preliminary assembly were closed using GapCloser. In addition, same-lane contamination was removed by filtering out reads with a sequencing depth of less than 0.35× of the average depth, yielding the final assembly. Fragments shorter than 500 bp were filtered out prior to statistical analysis and subsequent gene prediction.

#### 2.5.2 Genomic composition and functional analysis

Coding sequences in the newly sequenced genomes were predicted using GeneMarkS (Version 4.17) (http://topaz.gatech.edu/GeneMark/). Genomic islands were identified using IslandPath-DIMOB software (version 0.2). Predicted protein sequence were compared with the Non Redundant Protein Database (NR, https://blast.ncbi.nlm.nih.gov, accessed on August 10, 2024), and functional annotations were assigned using Gene Ontology (GO; http://geneontology.org/, accessed on August 10, 2024) and the Kyoto Encyclopedia of Genes and Genomes (KEGG, http://www.genome.jp/kegg On August 10, 2024). A database comparison was performed to analyze the metabolic pathways and functions of gene products and compounds in cells. After DIAMOND comparison (E-value ≤ 1e-5), the highest score comparison result (default identity ≥ 40%, coverage ≥ 40%) was selected for gene function annotation. Virulence factor genes were identified by aligning amino acid sequences against the Virulence Factors of Pathogenic Bacteria Database (VFDB, https://www.mgc.ac.cn/VFs/main.htm, On August 10, 2024) using DIAMOND. Antibiotic resistance genes were annotated using the Resistance Gene Identifier (RGI) by aligning amino acid sequences against the Comprehensive Antibiotic Resistance Database (CARD, https://card.mcmaster.ca/, Accessed on August 10, 2024) (RGI built-in BLASTP, default evaluation ≤ 1e-30) (Elnar and Kim, 2021; Jiang et al., 2022). For comparative analysis, whole genomes of *C. perfringens* from cattle, yaks, sheep, goats, and humans were downloaded from the National Center for Biotechnology Information (NCBI; https://www.ncbi.nlm.nih.gov, accessed on April 2, 2025) (**Table 2**). Multilocus sequence typing (MLST) was performed using the public databases for multi-locus sequence typing and microbial genome diversity (PubMLST; https://pubmlst.org/organisms, accessed April 19, 2025). Core-genome-based phylogenetic analysis was conducted using the Center for Genomic Epidemiology (https://cge.food.dtu.dk/services/CSIPhylogeny/, accessed on April 25, 2025) and CHiPlot (https://www.chiplot.online/tvbot.html, accessed on April 25, 2025).

**Table 2:** Information on *Clostridium perfringens* strains from the national center for biotechnology information.

Reference strain	ASM4605895v1	ASM4339461v1	ASM4857275v1	ASM1971087v1	ASM4193055v1	ASM3632148v1
Source	China Guizhou	China Nanjing	China Sichuan	China Shaanxi	China Chongqing	China Shaanxi
Animal	goat	cattle	yak	sheep	Human	dog
Genome size	3.6 MB	3.3 MB	3.5 MB	3.3 MB	3.3 MB	3.5 MB

## 3 Results

### 3.1 Incidence and clinical symptoms

**Figure 1:**
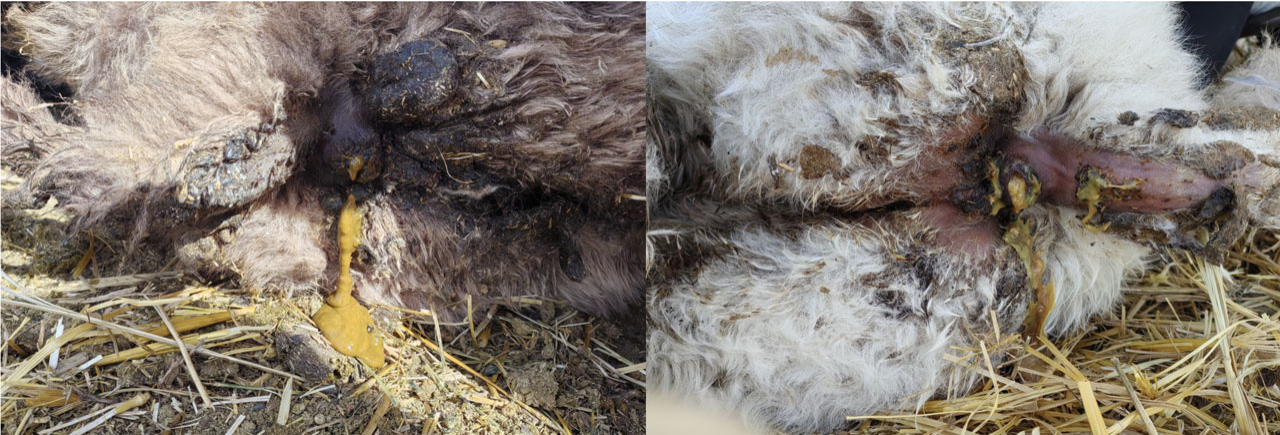
Yellow feces from a diseased young camel.

Over a 20-day period, 72 of 190 young camels on an Altay breeding farm developed the disease, resulting in an incidence rate of 37.89%. All affected camels were younger than 60 days old. Among these, 24 cases were acute, with animals collapsing and dying suddenly without prior symptoms, representing 12.63% of the total cases. These camels were in good nutritional condition. Additionally, 48 camels exhibited chronic attacks, and 15 died, corresponding to a chronic incidence of 25.26% and a fatality rate of 20.83%. Clinical signs included depression, complete anorexia, standing or lying with the head bent, cold ears and nose, and labored breathing. The oral mucosa changed progressively from pink to dark red to black-purple. Other symptoms included abdominal distension, diarrhea, and passage of yellowish or dark red porridge or watery malodorous feces (**Figure 1**). Neurological signs such as teeth grinding, muscle tremors, and weakness or slipping of the limbs often preceded death.

### 3.2 Physiological index measurement results

**Figure 2:**
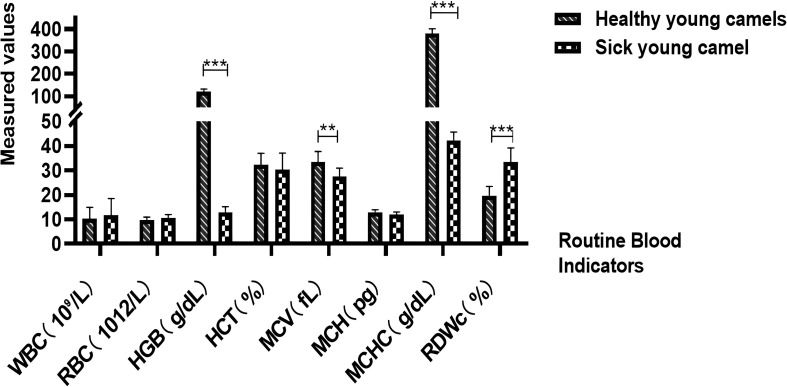
Blood routine analysis results of healthy and diseased young camels. ** indicates significant between-group differences (p < 0.05); *** indicates extremely significant between-group differences (p < 0.01). WBC: white blood cell, RBC: red blood cell, HGB: hemoglobin, HCT: hematocrit, MCV: mean corpuscular volume, MCH: mean hemoglobin concentration, MCHC: mean corpuscular hemoglobin concentration, RDWc: red cell distribution width.

**Figure 3:**
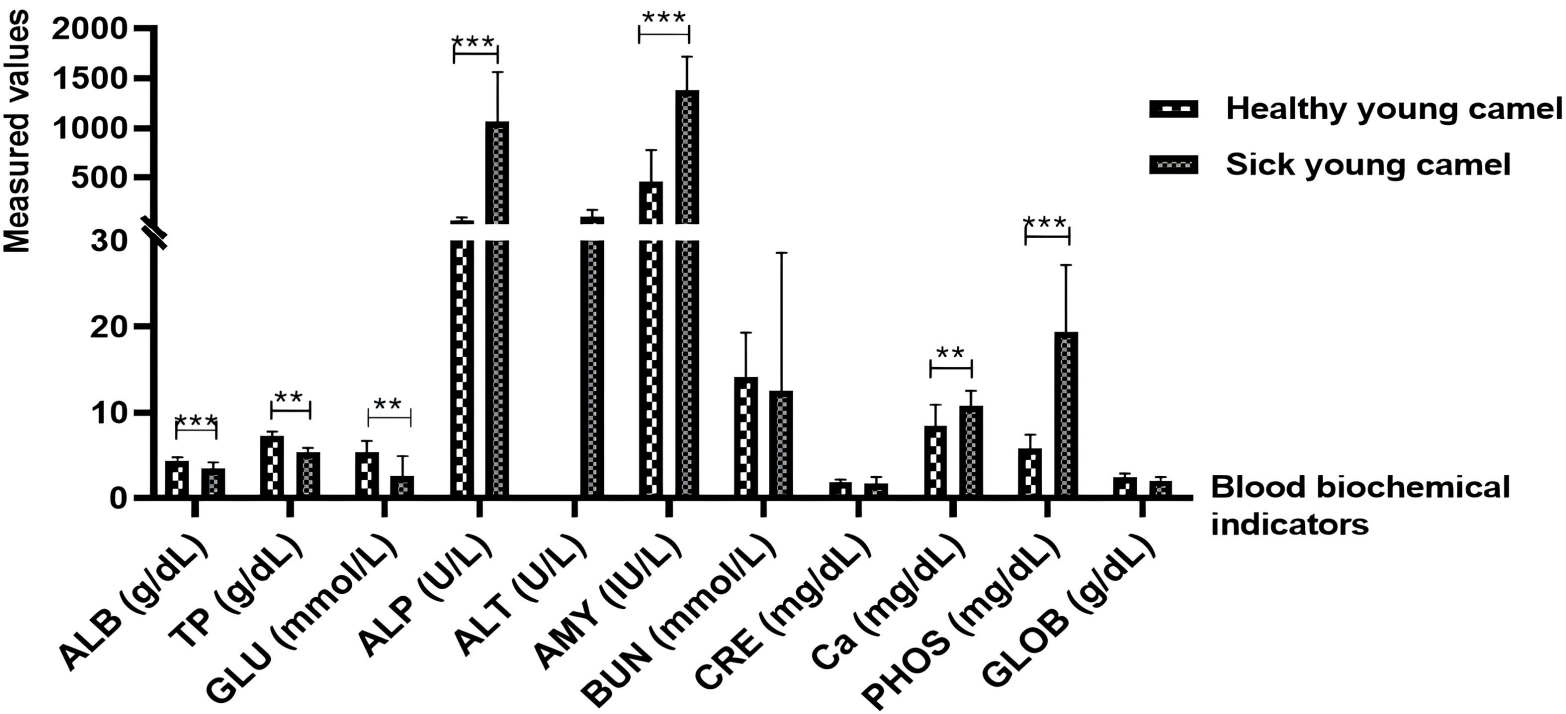
Blood biochemical analysis results of healthy and diseased young camels. ** indicates significant between-group differences (p < 0.05); *** indicates extremely significant between-group differences (p < 0.01). ALB: albumin, TP: total protein, GLU: glucose, ALP: alkaline phosphatase, ALT: alanine aminotransferase, AMY: amylase, BUN: blood urea nitrogen, CRE: creatinine, Ca: calcium, PHOS: phosphorus, GLOB: globulin (GLOB) levels.

The infected camels exhibited significantly higher body temperature, respiratory rate, and pulse than the healthy camels (**Table 3**). Routine blood test results revealed an increase in the white blood cell (WBC) and red blood cell (RBC) count, hemoglobin (HGB) levels, and red cell distribution width (RDWc), alongside reductions in the hematocrit (HCT) value, mean corpuscular volume (MCV), mean HGB concentration (MCH), and mean corpuscular HGB concentration (MCHC), with HGB and RDWc exhibiting particularly significant differences (**Figure 2**; **Supplementary Table 1**). Biochemical test results revealed decreased albumin (ALB), total protein (TP), glucose (GLU), blood urea nitrogen (BUN), creatinine (CRE), and globulin (GLOB) levels, and increased alkaline phosphatase (ALP), alanine aminotransferase (ALT), amylase (AMY), calcium (Ca), and phosphorus (PHOS) levels. Among these, ALB, ALP, AMY, and PHOS varied significantly from those in healthy controls, with ALP and AMY showing the most pronounced elevation (**Figure 3**; **Supplementary Table 2**).

**Table 3:** Temperature, respiration, and pulse of diseased and healthy young camels.

Index	Health group (range)	Disease group (range)
Temperature (°C)	38.0 ± 0.4 (37.2–38.6)	39.5 ± 0.5 (38.7–40.1)
Pulse rate (beats/min)	64.0 ± 8.2 (26.0–78.0)	71.4 ± 8.1 (61.0–90.0)
Respiratory rate (breaths/min)	24.0 ± 5.9 (17.0–37.0)	30.4 ± 4.8 (24.0–37.0)

### 3.3 Necropsy of dead camels

**Figure 4:**
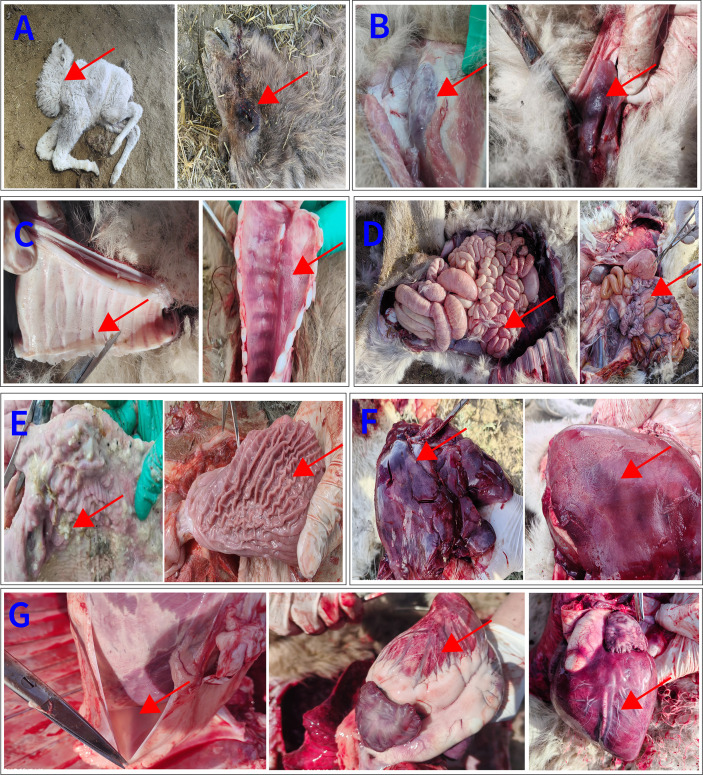
Necropsy observations of a dead camel. In the picture, **(A)** represents a dead camel, with arrows indicating head and neck curvature and eye hemorrhage, **(B)** represents the prescapular lymph nodes, with arrows indicating hemorrhage, **(C)** represents the trachea, with arrows indicating white exudate and hemorrhage. **(D)** represents the intestine, and the arrow indicates hemorrhagic gastroenteritis. **(E)** represents the rumen, and arrows indicate curd lumps and hemorrhage. **(F)** represents the liver, and the arrow indicates hemorrhage and a yellowish brown necrotic lesion. **(G)** represents the heart, and arrows indicate pericardial effusion.

**Figure 5:**
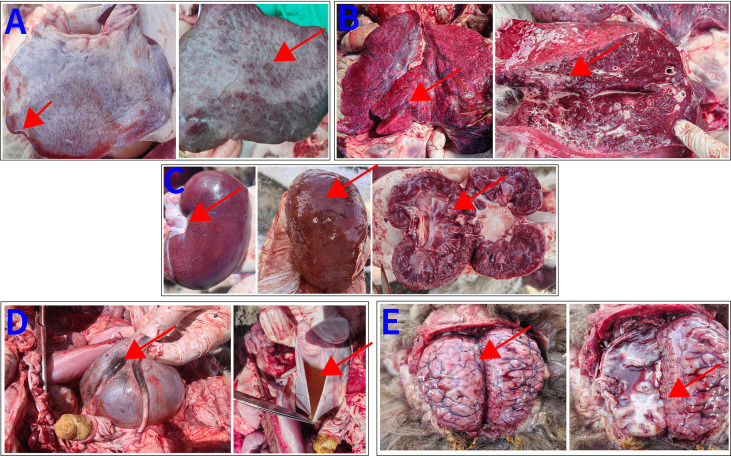
Necropsy observations of a dead camel. In the figure, **(A)** represents the spleen and the arrow represents hemorrhage. **(B)** represents the lungs, and arrows indicate lung lobe expansion and white exudate. **(C)** represents the kidney, with the arrow indicating liquefactive necrosis of the kidney, Coagulative necrosis of the renal papillae. **(D)** represents the bladder, arrows represent necrotic ureter and urine. **(E)** represents the brain, and arrows indicate edema and hemorrhage.

Necropsy revealed severe dehydration, with the head and neck bent and the abdomen markedly distended. The perianal area was contaminated with yellowish or black loose stool, and some camels exhibited ocular hemorrhage (**Figure 4A**). Enlarged prescapular lymph nodes with hemorrhages were observed (**Figure 4B**). The trachea exhibited congestion, containing abundant white foamy exudate (**Figure 4C**). The abdominal cavity contained pale-yellow fluid, and the intestinal mucosa displayed congestion and flushing, hemorrhage, and edema of the mesenteric lymph nodes. The intestines were gas-filled with thin and transparent walls, indicative of hemorrhagic enteritis (**Figure 4D**). The rumen exhibited mucosal edema and hemorrhage, with curd-like clumps in the stomach (**Figure 4E**). The liver was enlarged, with hemorrhagic, brittle texture, black-red color, and scattered yellow necrotic lesions of varying sizes on the surface (**Figure 4F**). Pericardial effusion, focal myocardial necrosis, petechiae, and dendritic vascular swelling were noted (**Figure 4G**). Additional findings included splenomegaly with hemorrhage (**Figure 5A**). The lungs of most of the dead camels adhered to the thoracic cavity and became dark, exhibiting expansion and congestion with foamy exudate in the bronchi (**Figure 5B**); the kidneys were enlarged, dark, congested, or hemorrhagic. Chronic cases exhibited cortical dissolution and coagulative necrosis of the renal papillae (**Figure 5C**). Some camels exhibited bladder and ureteral necrosis with dark yellow urine (**Figure 5D**). Meningeal congestion, edema, meningeal vasodilation, and hemorrhage were observed; chronic cases exhibited thick, milky white pia mater, brain edema, flattened gyri, and shallow sulci (**Figure 5E**).

### 3.4 Histopathological observation

**Figure 6:**
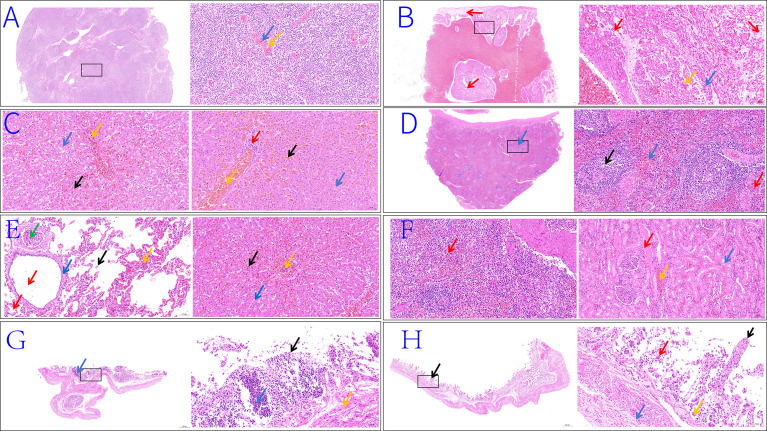
Histopathological findings in tissues from a deceased camel. **(A)** represents the prescapular lymph nodes, with orange arrows indicating vascular congestion and dilation, and blue arrows indicating pigment deposition. **(B)** represents the heart, the blue arrow represents muscle fiber atrophy, the red arrow represents lymphocyte infiltration, and the orange arrow represents congestion. **(C)** represents the liver, with blue arrows indicating edema, black arrows indicating hepatic sinusoid congestion, red arrows indicating lymphocyte infiltration, and orange arrows indicating vascular congestion. **(D)** represents the spleen, black arrows represent lymphocytes, blue arrows represent congestion, and red arrows represent granulocytes. **(E)** represents the lungs, orange arrows indicate capillary congestion, black arrows indicate granulocytes, red arrows indicate hemorrhage, blue arrows indicate flattening of bronchial epithelial cells, and green arrows indicate white blood cells. **(F)** represents the kidney, the blue arrow represents edema, the red arrow represents acidophilic substances, and the orange arrow represents vascular congestion. **(G)** represents the duodenum, black arrows represent shed intestinal villi, blue arrows represent macrophage infiltration, and orange arrows represent edema. **(H)** represents the ileum, the black arrow represents shed intestinal villi, the orange arrow represents shed intestinal glandular epithelium, the blue arrow represents edema, and the red arrow represents congestion.

Microscopic examination revealed extensive pathological changes across multiple organs. Lymphoid tissue showed densely packed, evenly distributed lymphocytes with numerous visible lymph nodes, marked interstitial vascular congestion and dilation (orange arrow in **Figure 6A**), occasional brownish-yellow pigment deposition, and no medulla formation (blue arrow in **Figure 6A**). Cardiac tissue exhibited marked atrial congestion. Atrial myocardial fibers were finer than those in the ventricles and were arranged loosely and irregularly. Mild muscle fiber atrophy was evident (blue arrow in **Figure 6B**), with widened intercellular spacing, interstitial multifocal edema, loose connective tissue, scattered lymphocytes (red arrow in **Figure 6B**), and capillary congestion (orange arrow in **Figure 6B**). Liver sections displayed indistinct lobular boundaries, with a central vein and a roughly radial arrangement of liver cells and surrounding hepatic sinusoids. Mild hepatocellular edema (blue arrow in **Figure 6C**), loose and pale cytoplasm, and a pronounced sinusoidal congestion and dilation (black arrow in **Figure 6C**) were also observed. Occasional mild lymphocyte infiltration was observed in the portal area (red arrow in **Figure 6C**), along with marked vascular congestion (orange arrow in **Figure 6C**). The splenic tissue membrane was composed of dense connective tissue rich in elastic and smooth muscle fibers of uniform thickness. This connective tissue extended inwards to form well-developed trabeculae without apparent abnormalities. The spleen comprised red and white pulp; the white pulp contained lymphatic sheaths, lymph nodes, and marginal areas around the central artery, with a clear structure, few cells, and regular shape. Lymphocytes were more commonly observed around the central artery in the white pulp, where they were sparse and loosely arranged (black arrow in **Figure 6D**). The red pulp occupied a large area beneath the capsule, around the trabeculae, and along the outer edge of the white pulp. The red pulp was composed of the splenic cord and sinusoids and had a clear boundary with the white pulp. It was widely congested (blue arrow in **Figure 6D**), with sparse and scattered granulocytes (red arrow in **Figure 6D**). Lung tissue was covered with smooth serosa without obvious abnormalities; the alveolar wall consisted of a single layer of epithelial cells with a clear structure. Capillaries exhibited multifocal congestion (orange arrows in **Figure 6E**), alveolar size varied, and small eosinophilic deposits were scattered in the alveolar cavity (black arrows in **Figure 6E**). Diffuse hemorrhage (red arrow in **Figure 6E**), with a small amount of red blood cells visible in the alveoli and bronchioles, occasional flattening of bronchial epithelial cells (blue arrow in **Figure 6E**), occasional cellular debris, unstructured eosinophils, and WBCs within the blood vessels were observed (green arrows in **Figure 6E**). The renal parenchyma consisted of a shallow cortex and deep medulla, with a clear boundary between the cortex and medulla. The distribution of glomeruli in the cortex was uniform, and the number of cells and matrix in the glomeruli were uniform. Edema was observed in a large number of renal tubular epithelial cells (blue arrow in **Figure 6F**). Additionally, loose and pale cytoplasm and a small amount of eosinophilic substances were detected in the renal tubules (red arrow in **Figure 6F**). No significant proliferation was observed in the renal interstitium (the connective tissue between the urinary tubules); however, marked interstitial vascular congestion (orange arrow in **Figure 6F**) was noted, with no obvious inflammatory cell infiltration. Widespread shedding of intestinal villi was observed in the duodenum (black arrow in **Figure 6G**), the lamina propria was exposed, the structure of intestinal glands was indistinct, and focal infiltration of lymphocytes and macrophages was observed (blue arrow in **Figure 6G**). The submucosa revealed mild edema (orange arrow in **Figure 6G**), loose connective tissue, occasional lymphocyte infiltration, uneven muscle layer thickness, and an irregular muscle fiber arrangement. Widespread shedding of intestinal villi was detected in the ileum (black arrow in **Figure 6H**), with an exposed lamina propria and a loose arrangement of intestinal glands. A pronounced amount of intestinal gland epithelium was shed, with increased distance between the villi and the basement membrane (orange arrow in **Figure 6H**), and the submucosal layer contained loose connective tissue, uneven thickness of the muscle layer, multifocal edema, and loose arrangement of muscle fibers and connective tissue, accompanied by lymphocyte infiltration (blue arrow in **Figure 6H**) and massive vascular congestion (red arrow in **Figure 6H**).

**Figure 7:**
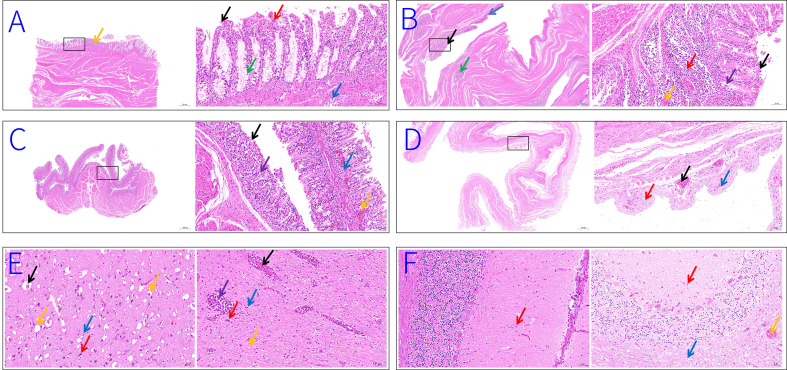
Histopathological findings in tissues from a deceased camel. **(A)** represents the cecum, the black arrow represents the shed intestinal mucosa, the green arrow represents the necrotic intestinal gland, the orange arrow represents a small area of unclear intestinal gland structure, the red arrow represents congestion, and the blue arrow represents macrophage infiltration. **(B)** represents the rumen, the blue arrow represents loose and lightly stained cytoplasm of epithelial cells, the black arrow represents shed mucosal epithelium, the purple arrow represents shed gastric gland epithelium, the red arrow represents macrophage infiltration, the orange arrow represents congestion, and the green arrow represents lymphocytes. **(C)** represents the reticulum, with black arrows indicating shed mucosal epithelium, blue arrows indicating loose and lightly stained cytoplasm of gastric glandular epithelial cells, purple arrows indicating shed gastric glandular epithelium, and orange arrows indicating congestion. **(D)** represents the bladder, the blue arrow represents edema, the red arrow represents loose and lightly stained cytoplasm, and the black arrow represents congestion. **(E)** represents the brain, the red arrow represents atrophied neurons, the blue arrow represents distributed glial cells, the orange arrow represents a small vacuole, the black arrow represents congestion, and the purple arrow represents macrophage infiltration. **(F)** represents the cerebellum, the red arrow represents fewer glial cells, the blue arrow represents small bubble, and the orange arrow represents congestion.

The cecal epithelium was extensively shed and incomplete (black arrow in **Figure 7A**). Numerous short, tubular, densely arranged intestinal glands were observed in the lamina propria. Frequent glandular necrosis was observed (green arrow in **Figure 7A**), characterized by nuclear condensation, cytoplasmic disintegration, and localized structural loss (orange arrow in **Figure 7A**), along with marked vascular congestion (red arrow in **Figure 7A**). The muscularis mucosa separated the lamina propria from the submucosal layer, which was composed of loose connective tissue, a thick muscle layer, irregular muscle fibers, and a focal infiltration of macrophages (blue arrow in **Figure 7A**). In the rumen, the non-glandular region was lined with stratified squamous epithelium, where some epithelial cells exhibited loose, lightly stained cytoplasm (blue arrow in **Figure 7B**). Extensive mucosal shedding was evident in the glandular area (black arrow in **Figure 7B**), with abundant, tightly packed gastric glands in the lamina propria and minor epithelial loss (purple arrow in **Figure 7B**). Numerous lymphocytes and macrophages infiltrated locally (red arrow in **Figure 7B**), accompanied by severe vascular congestion (orange arrow in **Figure 7B**), uneven muscle thickness, irregular fiber arrangement, and occasional focal lymphocyte and macrophage aggregation (green arrow in **Figure 7B**). The mucosal layer of the reticulum displayed prominent mucosal folding into the gastric cavity and widespread mucosal epithelial shedding (black arrow in **Figure 7C**), exposing the lamina propria with numerous gastric glands. Many gastric glandular epithelial cells had loose and lightly stained cytoplasm (blue arrow in **Figure 7C**), and minor epithelial loss widened the basement membrane gap (purple arrow in **Figure 7C**). A large amount of vascular congestion (orange arrow in **Figure 7C**) was also observed. The submucosal layer was composed of loose connective tissue, a thick muscle layer, and irregular muscle fibers. Bladder tissue exhibited intact mucosal epithelium with edematous epithelial cells (blue arrow in **Figure 7D**), loose and lightly stained cytoplasm, and clustered chromatin edges in the nucleus (red arrow in **Figure 7D**). Mild vascular congestion occurred in the lamina propria, with few leukocytes in the blood vessels (black arrow in **Figure 7D**). Muscle layers were uneven and irregularly arranged. Brain tissue revealed mildly shrunken neurons (red arrow in **Figure 7E**), reduced cell volume, irregular shape, unclear nuclear cytoplasmic boundaries, deepened staining, and abundant scattered glial cells (blue arrow in **Figure 7E**). Numerous small vacuoles were observed (orange arrows in **Figure 7E**), accompanied by severe vascular congestion (black arrow in **Figure 7E**) and mild perivascular lymphocyte and macrophage infiltration into a ring, forming perivascular sleeves (purple arrow in **Figure 7E**). Cerebellar tissue was covered with meninges without obvious abnormalities. The cerebellar cortex showed clear molecular, Purkinje cell, and granular layers, with fewer glial cells in the molecular layer (red arrow in **Figure 7F**) and fewer cells in the granular layer; the medullary tissue was arranged loosely, with numerous small vacuoles (blue arrows in **Figure 7F**) and marked vascular congestion (orange arrows in **Figure 7F**).

### 3.5 Isolation and identification of bacteria and 16S rRNA gene sequence analysis

**Figure 8:**
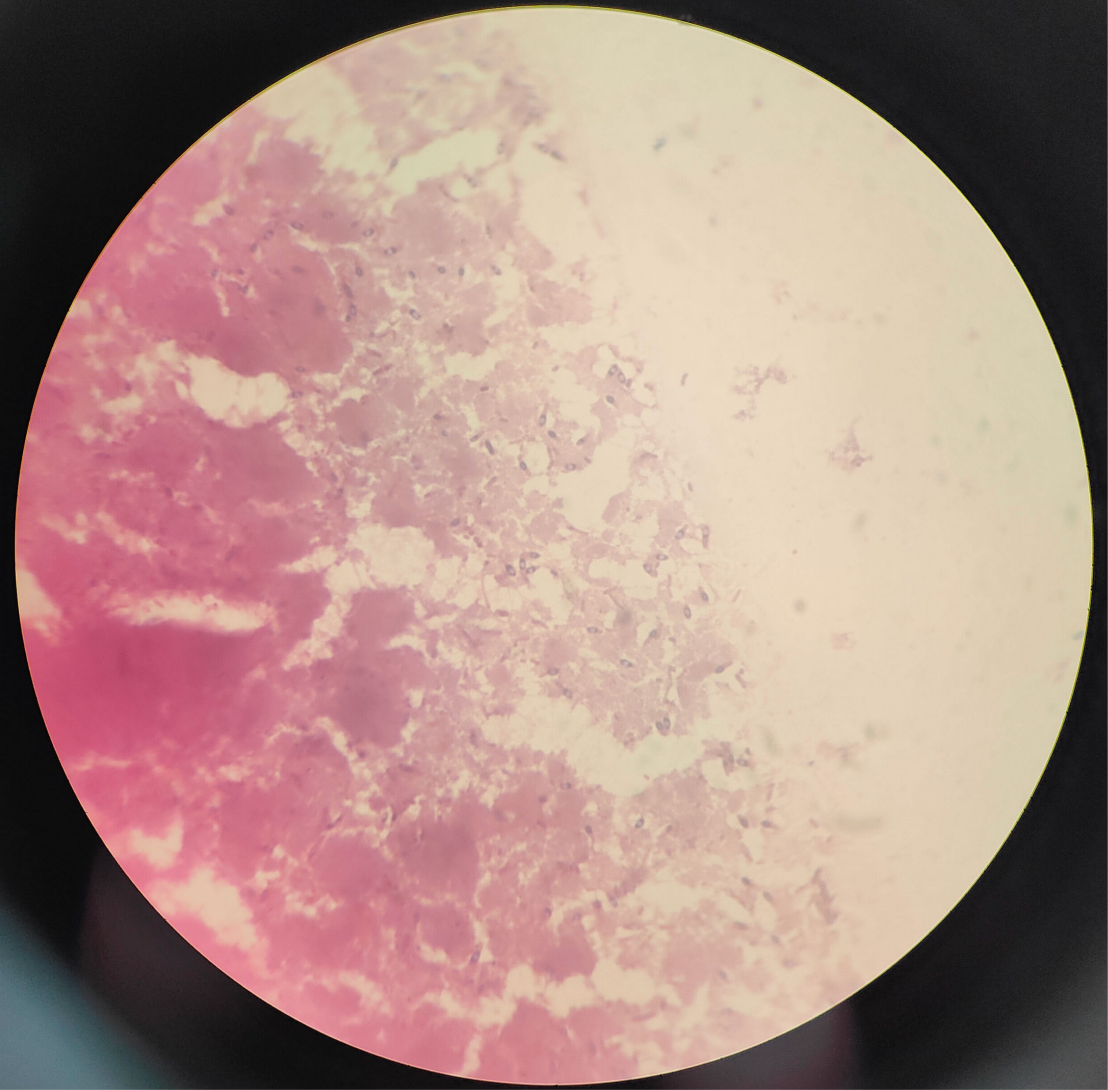
Gram staining of liver tissue (100×).

**Figure 9:**
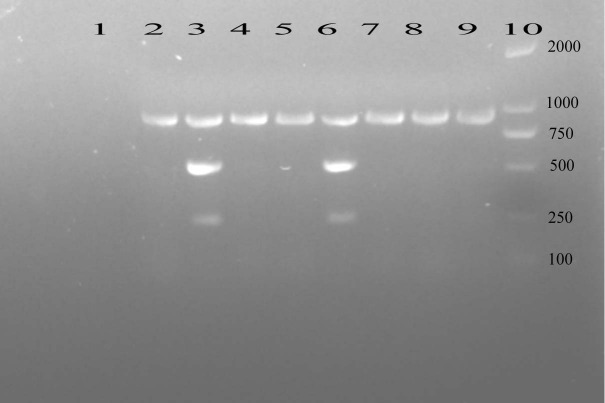
Multiplex polymerase chain reaction (PCR) detection results of isolated *Clostridium perfringens.* Lane 1: blank control. The *plc* (fragment size: 900 bp) gene was detected in all eight strains of bacteria (lanes 2–9), while the *etx* (fragment size: 396 bp) and *cpe* (fragment size: 233 bp) genes were detected in lanes 3 and 6. Lane 10: DNA marker.

Gram-stained liver tissue slides revealed numerous Gram-positive bacteria, forming round or oval spores with a diameter larger than that of the bacterial cells, located centrally, terminally, or subterminally, with spindle-shaped cell expansion (**Figure 8**). The eight isolated bacteria formed round or oval black colonies on the TSC agar, surrounded by milky white opaque rings, with smooth, moist surfaces and neat edges (**Supplementary Figure 1**). Gram staining of single colonies exhibited blunt-ended rounded positive rods at both ends, occurring singly or in pairs, without flagella (**Supplementary Figure 2**). After 2 h of milk fermentation, all strains exhibited vigorous fermentation, producing sponge-like curds that floated to the surface of the culture medium after crushing (**Supplementary Figure 3**). The dynamic nitrate reduction test revealed that all the strains diffused and grew along the puncture line and reduced nitrate to nitrite, as evidenced by the red coloration of the medium (**Supplementary Figure 4**). Lactose gelatin puncture indicated gas production and acid formation, with the medium changing from red to yellow; gelatin remained solid after 1 h at 5°C (**Supplementary Figure 5**). PCR analysis detected *plc* genes in all eight strains, while *etx* and *cpe* genes were present in two strains (**Figure 9**), and *cpb*, *iap*, *cpb2*, and *netB* genes were absent. At the same time, the 16S rRNA gene sequencing results were compared using NCBI-BLAST, confirming once again that the isolated strains were all *C. perfringens*.

### 3.6 Bacterial gene assembly and genomic analysis

Whole-genome sequencing revealed that the average read lengths of JLFS-2 and JLFS-7 were 1, 168.27 bp and 13, 116.5 bp, respectively, and that the N50 read lengths were 12, 007.0 bp and 14, 252.0 bp, respectively (**Supplementary Table 3**). JLFS-2 had a genome size of 3, 372, 358 bp comprising 3, 099 predicted coding genes. It consisted of one chromosome and three circular plasmids. The chromosome was 3, 274, 258 bp in length, accounting for 97.09% of the genome, while the circular plasmid sequences measured 49, 444 bp, 38, 621 bp, and 10, 008 bp, respectively. In addition, the genome contained 96 tRNA genes and 10 rRNA genes (5S, 16S, and 23S). JLFS-7 had a total genome length of 3, 583, 661 bp with 3, 409 predicted coding genes. It included one chromosome and two circular plasmids with a chromosome sequence length of 3, 485, 296 bp, accounting for 97.25% of the genome. The circular plasmid sequences were 88, 264 bp and 10, 101 bp, respectively. A total of 94 tRNA genes and 10 rRNA genes (5S, 16S, and 23S) were identified (**Table 4**; **Supplementary Table 3**). For the remaining six strains, the clean reads ranged from 1, 202.81 M to 2, 008.60 M. Among the assembled contigs longer than 500 bp, the total number ranged from 14 to 16, the N50 length ranged from 239, 542 bp to 2, 038, 063 bp, and the GC content ranged from 28.10% to 28.26%, The genome sizes ranged from 2.57 Mb to 5.23 Mb, with total sequence lengths ranging from 3, 195, 592 bp to 3, 595, 833 bp. The number of predicted protein-coding genes ranged from 2, 897 to 3, 413, and the genome also included 88–94 tRNAs, along with 0–10 copies of rRNA genes (5S, 16S, and 23S) and sRNA genes (**Supplementary Table 3**).

**Table 4:** Statistical table of prediction results for coding genes.

Sample ID	Genome size (bp)	Gene number	Gene total length (bp)	Gene average length (bp)	Gene length/genome (%)
JLFS-1	3, 461, 394	3, 244	2, 915, 367	899	84.23
JLFS-2	3, 372, 358	3, 099	2, 824, 011	911	83.74
JLFS-3	3, 294, 466	3, 008	2, 780, 478	924	84.4
JLFS-4	3, 595, 833	3, 415	3, 032, 751	888	84.34
JLFS-5	3, 346, 911	3, 073	2, 824, 227	919	84.38
JLFS-6	3, 296, 269	3, 005	2, 776, 422	924	84.23
JLFS-7	3, 583, 661	3, 409	3, 008, 856	883	83.96
JLFS-8	3, 195, 592	2, 897	2, 692, 731	929	84.26

JLFS-2 and JLFS-7 harbored 9 and 11 genomic islands, totaling 100, 318 bp and 107, 252 bp, respectively. The remaining six strains were predicted to contain 3–11 virulence islands with a total length ranging from 27, 692 bp to 84, 253 bp (**Table 4**; **Supplementary Table 3**). Clustered regularly interspaced short palindromic repeats (CRISPR) are a unique DNA sequence family widely present in prokaryotic genomes (Zhang et al., 2024). CRISPR predictions for the genomes of the eight strains revealed that JLFS-3, JLFS-5, and JLFS-8 lacked predicted CRISPR arrays, whereas the total number of predicted CRISPR arrays for the remaining strains ranged from 1 to 4 (**Table 5**).

**Table 5:** Clustered regularly interspaced short palindromic repeats prediction results.

Sample_ID	CRISPR_num	Total_length	Average_length
JLFS-1	3	1, 552	517.333
JLFS-2	4	1406	351.5
JLFS-4	2	540	270
JLFS-6	1	164	164
JLFS-7	1	359	359

### 3.7 Gene function annotation analysis

**Figure 10:**
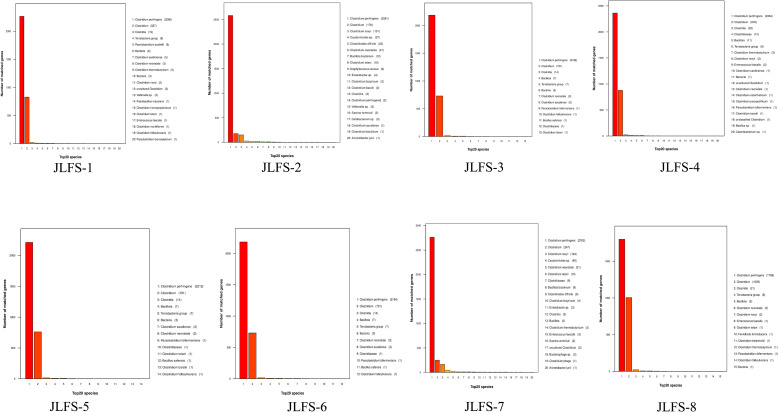
Functional classification predicted genes based on the non-redundant protein database annotation in different *Clostridium perfringens* strains. The x-axis represents the species ID, and the y-axis indicates the number of annotated genes.

**Figure 11:**
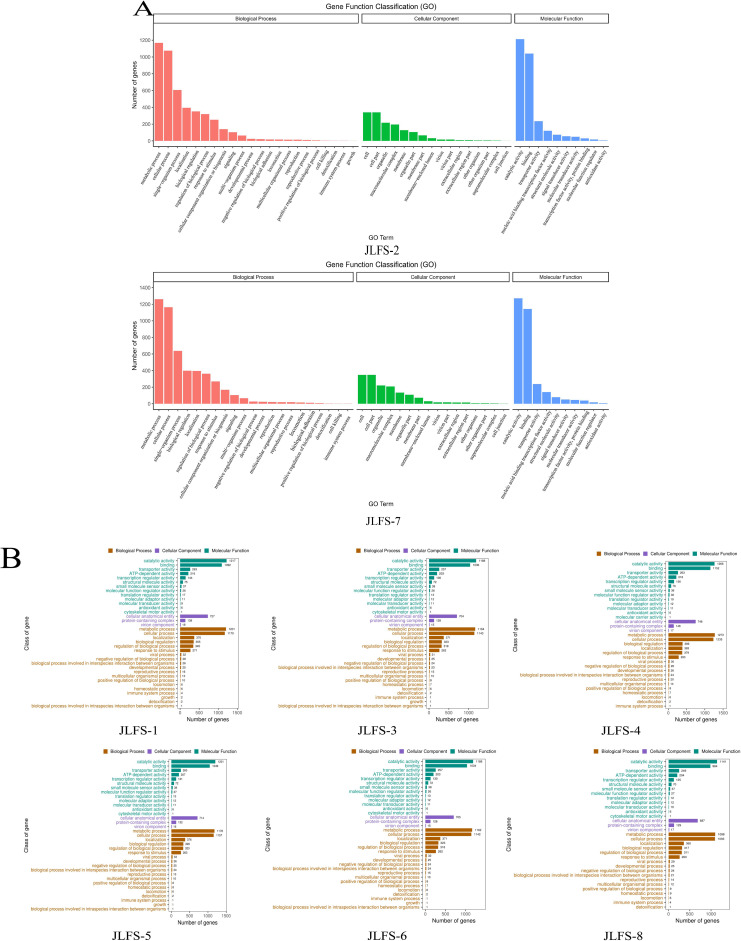
Functional classification of predicted genes based on the gene ontology (GO) annotation for each sample. **(A)** The x-axis represents the GO term at the next level for the three main GO categories, and the y-axis indicates the number of genes annotated to each term. **(B)** The x-axis shows the number of annotated genes, and the y-axis represents the level 2 functional classification of GO on the sample gene annotation.

Annotation against the NR database confirmed that all eight strains primarily corresponded to *C. perfringens* (**Figure 10**). GO analysis revealed that the strain JLFS-2 was annotated with 4, 605, 1, 439, and 2, 839 genes under biological processes, cellular components, and molecular functions, respectively, while the strain JLFS-7 was annotated with 4, 969, 1, 548, and 3, 029 genes under biological processes, cellular components, and molecular functions, respectively (**Figure 11A**). Across all strains, the dominant biological process terms were metabolic and cellular processes, whereas the molecular function terms were mainly catalytic activity and binding. For cellular components, JLFS-2 and JLFS-7 were enriched in cell and cell part categories; other strains demonstrated annotations for cellular anatomical entities and protein-containing complexes, likely reflecting differences in sequencing depth (**Figure 11B**).

**Figure 12:**
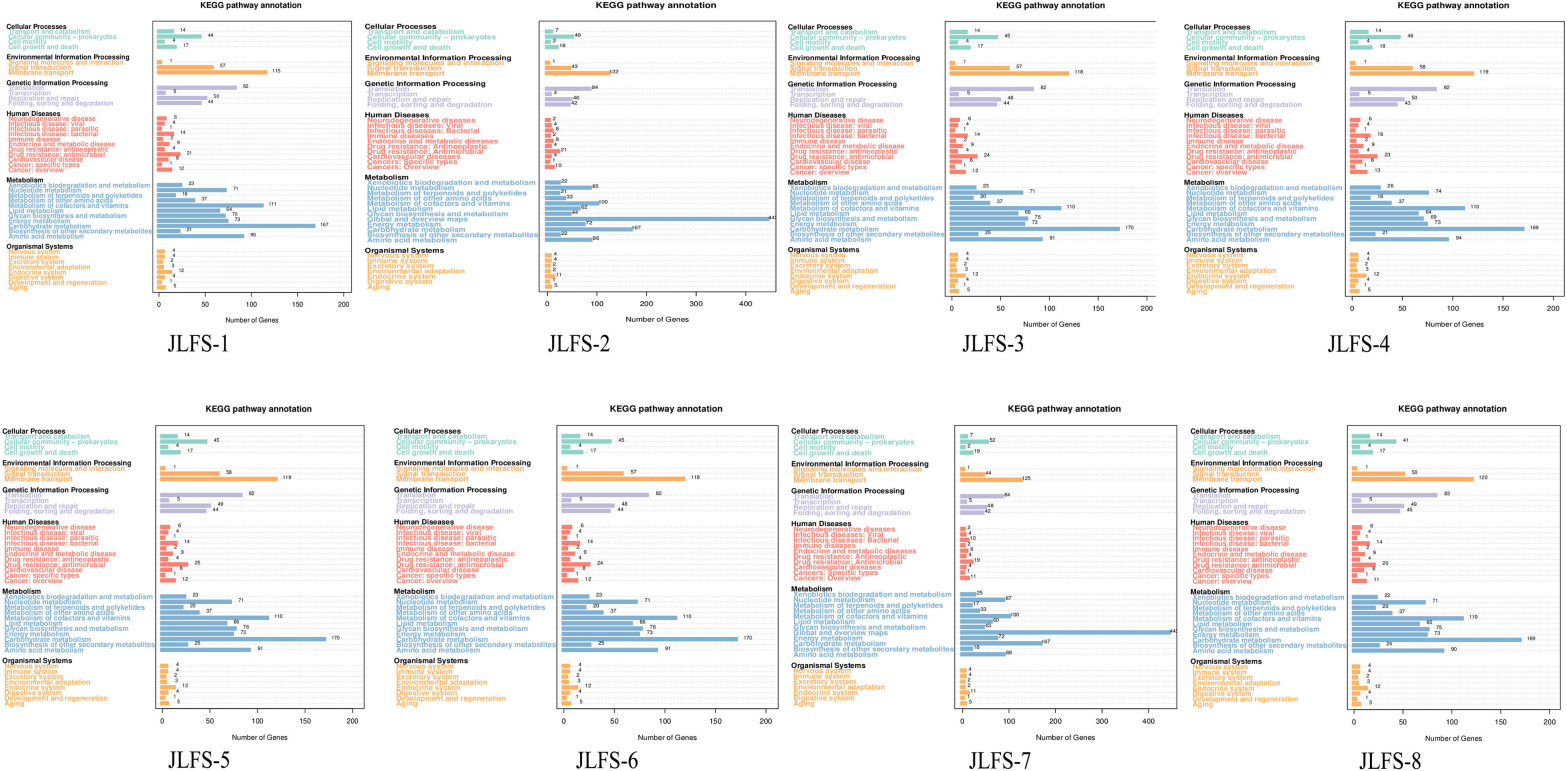
Classification of metabolic pathways based on the Kyoto Encyclopedia of Genes and Genomes (KEGG) annotation of predicted genes. The x-axis represents the number of annotated genes. The y-axis represents the annotated metabolic pathways.

KEGG metabolic pathway classification indicated that 2, 411 genes of the JLFS-2 strain were annotated to 189 KEGG metabolic pathways, 2, 419 genes of the JLFS-7 strain were annotated to 187 KEGG metabolic pathways, and other strains were annotated to 188–200 pathways, with 1, 553–1, 579 genes (**Supplementary Table 4**). The metabolic pathways of the eight bacterial strains were mainly associated with six KEGG functional categories, including metabolism, genetic information processing, environmental information processing, cellular processes, human diseases, and organismal systems. Between 56.40% and 70.38% of genes were involved in metabolism, with the main metabolic pathways being global and overview maps and carbohydrate metabolism. Between 4.62% and 6.63% of genes were involved in cellular processes, with the main metabolic pathways being cellular community–prokaryotes and cell growth and death. Between 10.71% and 14.09% of genes were involved in genetic information processing, with the main metabolic pathways being translation and replication and repair. Between 3.89% and 13.54% of the genes were involved in environmental information processing, with the main metabolic pathways being signaling molecules and interaction and signaling transduction. Approximately 6.45% of the genes were involved in human diseases, with the main metabolic pathways being drug resistance (antimicrobial) and infectious diseases (bacterial). Between 1.74% and 2.72% of genes were involved in organismal systems, with the main metabolic pathways being the endocrine system and aging (**Figure 12**).

### 3.8 Construction of a phylogenetic tree based on core genome and MLST typing

**Figure 13:**
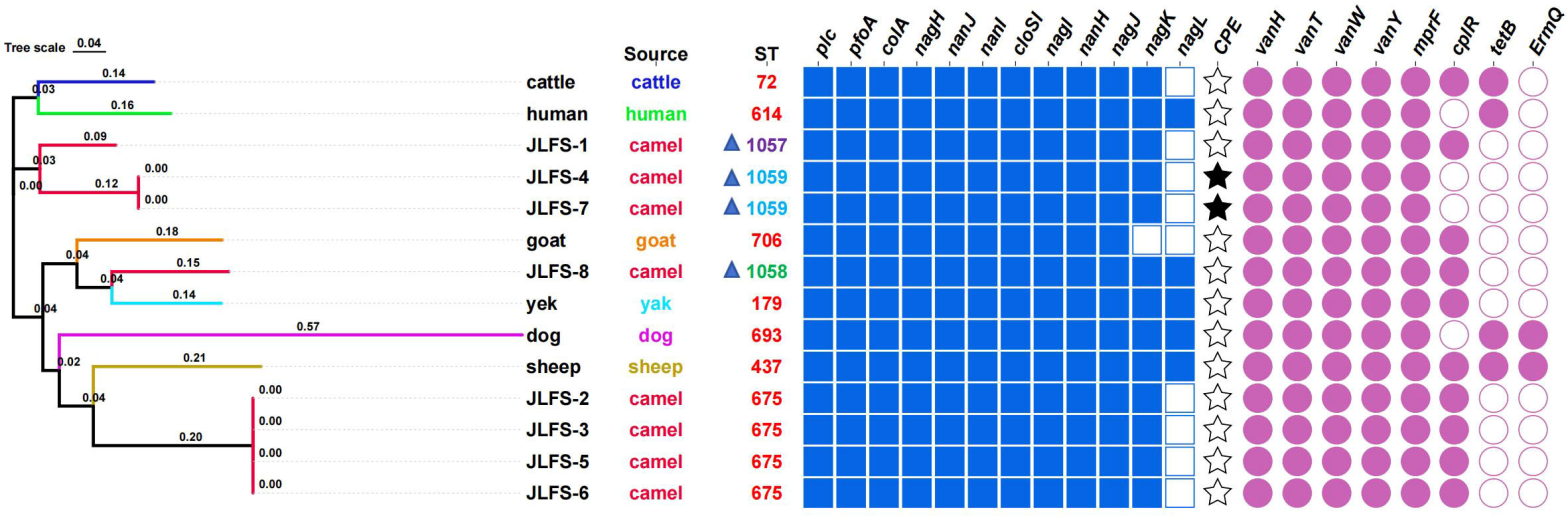
Phylogenetic tree based on core genome. The triangle in the figure represents the newly discovered ST type, and the black five pointed star represents the core gene.

VFDB identified 13 virulence genes encoding seven toxins: alpha toxin (*plc*), theta toxin (*pfoA*), kappa toxin (*colA*), mu toxin (*nagH, nagI, nagJ, nagK, and nagL*), sialidase (*nanJ, nanI, and nanH*), alpha clostridial protease (*cloSI*), and enterotoxin (*cpe*) genes. JLFS-4 and JLFS-7 were annotated as containing *cpe* genes, whereas *nagL* was detected only in the JLFS-8 strain (**Figure 13**).

CARD analysis revealed six antimicrobial resistance genes across all isolates: *vanH, cpIR, vanT, vanW, vanY*, and *mprF*. However, the *cpIR* gene was absent in the JLFS-4 and JLFS-7 strains, which may indicate variable resistance profiles. According to the antibiotic annotation results of different genes based on the CARD database, these strains may be resistant to antibiotics such as lincomycin, clindamycin, virginiamycin M1, retapamulin, iboxamycin, vancomycin, teicoplanin, and defensin (**Supplementary Table 4**). Reference strains additionally annotated *ErmQ* and *tetB* resistance genes, with the resistance types of the sheep and dog reference strains being significantly higher than those of the other strains (**Figure 13**). Overall, *C. perfringens* isolates from Bactrian camels harbored fewer resistance genes, possibly reflecting host-specific ecological constraints.

The sequencing results were compared against the PubMLST database sequence analysis, and four ST types were detected among the isolates, including three newly discovered types: 1, 057, 1, 058, and 1, 059 (**Table 6**). The dominant ST among Bactrian camel *C. perfringens* isolates was ST675, accounting for 50% of the total. No common ST was found between Bactrian camels and other animals, suggesting limited evidence for cross-host transmission. Phylogenetic analysis of 13 strains of *C. perfringens* strains demonstrated that strains JLFS-2, JLFS-3, JLFS-5, and JLFS-6 exhibited high homology with sheep, whereas strain JLFS-8 showed the greatest similarity to yak. In contrast, JLFS-1, JLFS-4, and JLFS-7 strains, which represent newly identified ST types, displayed substantial differences in homology with other animal strains of *C. perfringens*, confirming that *C. perfringens* isolates from Bactrian camels may follow a distinct evolutionary trajectory (**Figure 13**).

**Table 6:** New multilocus sequence typing types of *Clostridium perfringens*.

Strain number	Source	Sequence types	colA	groEL	sodA	plc	gyrB	sigk	Pgk	nadA
JLFS-1	Camel	1057	66	5	1	50	1	5	4	1
JLFS-4	Camel	1059	3	5	1	1	7	7	5	1
JLFS-7	Camel	1059	3	5	1	1	7	7	5	1
JLFS-8	Camel	1058	4	1	3	156	1	1	2	46

## 4 Discussion

According to statistics from the Food and Agriculture Organization of the United Nations, camels provide a vital source of livelihood for millions of families in arid and mountainous regions worldwide, contributing significantly to reducing hunger, eradicating poverty, and achieving sustainable development of land ecosystems (Abu-Seida et al., 2024). In 2023, the Bactrian camel population in China reached approximately 561, 800, primarily distributed in the deserts and Gobi of Xinjiang, Inner Mongolia, Gansu, and other regions, with regional stocks of 315, 600, 206, 300, and 39, 900, respectively (Yao et al., 2023; Ma et al., 2025b). However, the rapid expansion of large-scale breeding has coincided with an increase in *C. perfringens* infections, posing serious health and economic challenges (Al-Ruwaili et al., 2012; Fayez et al., 2013; Hm et al., 2024; Ma et al., 2025a). Despite its impact, research on *C. perfringens* in Bactrian camels remains limited, and no genome-wide studies have been reported until now.

Our study revealed that *C. perfringens* disease in Bactrian camels predominantly affects young camels, with an incidence of 37.89%, which is lower than the 56.3% reported in Saudi Arabia (Fayez et al., 2020). Acute mortality accounted for 12.63%, also lower than the 15% in eastern Saudi Arabia (Mubarak et al., 2024). *C. perfringens* disease in Bactrian camels typically occurred in camels younger than 2 months, while the disease in single-humped camels occurred mainly in animals older than 2 years (Mubarak et al., 2024). Clinical signs, physiological indicators, autopsies, and histopathological examinations are essential references for disease diagnosis (Hussain et al., 2022; Heidari et al., 2023). Similar to dromedaries, Bactrian camels experience diarrhea, recumbency, and neurological signs. Necropsy findings include foam-like tracheal exudates, adherence of lung to the pleura, hemorrhage and swelling in multiple organs, such as heart, liver, spleen, and kidney, intestinal hemorrhage, congestion, and flatulence (Fayez et al., 2013; Hm et al., 2024). Clinical signs include increased body temperature, respiratory rate, and pulse, with pulse reaching up to 90 bpm, reflecting disease stage, inflammation severity, and heart function abnormalities. Moreover, increased respiratory rate indicated that the lungs had developed lesions (Quinton et al., 2018). Hematological analysis revealed elevated WBC, RBC, HGB, and RDWc, alongside reduced HCT, MCV, MCH, and MCHC, reflecting inflammatory responses (Kong et al., 2021). In parallel, diseased camels exhibited symptoms such as dehydration, hypoxia, and anemia (Martín-Barrasa et al., 2023). The average values of WBC (10.3 ± 4.6 x 10 ^9^06.3 RBC (9.8 ± 1.1 x 10 ¹²/L), MCV (33.4 ± 4.3 fL), MCH (12.9 ± 1.1 pg), and MCHC (379.4 ± 21.3 g/dL) measured in healthy young camels in our study were higher than those reported by El Sayed AA et al (El-Sayed et al., 2025) in adult unimodal camels (WBC (14.4 ± 3.8 x 10^3^/L), RBC (11.4 ± 1.6 x 10^6^/L), MCV (28.3 ± 3.7 fL), MCH (12.4 ± 2.9 pg), and MCHC (40.5 ± 8.1 g/dL)). This difference may be attributed to animal age and type of breeding (Martín-Barrasa et al., 2023; El-Sayed et al., 2025). The levels of ALB, TP, GLU, BUN, CRE, and GLOB in diseased camels were reduced. ALP, ALT, AMY, and PHOS levels were increased, reflecting significant inflammation or damage to the liver and kidneys of affected young camels (Shimada and Mitchison, 2019; Baker et al., 2024). Serum TP and albumin can be used to assess the nutritional status of animals. The TP and albumin levels in affected camels were lower than those measured in healthy young camels, which may be related to nutritional consumption and decreased feeding of affected camels (Amin et al., 2007). The ALB (3.38 ± 0.47 g/dL), TP (5.56 ± 0.56 g/dL), and BUN (10.60 ± 6.08 mg/dL) values of the Canary Islands Bactrian camels were lower than those measured in healthy young camels (Martín-Barrasa et al., 2023); however, the ALB (4.57 ± 0.77 g/dL) and BUN values of Indian Bactrian camels (Navarro et al., 2020; Zhu et al., 2025) were lower than those of healthy young camels (Martín-Barrasa et al., 2023). The biochemical parameters of Bactrian camels revealed notable differences between diseased and healthy juveniles. In Indian Bactrian camels, glucose was 57 ± 10.19 mg/dL and GLOB was 3.84 ± 0.63 g/dL, both higher than values reported for healthy juveniles (De et al., 2020). In Canary Islands Bactrian camels, AMY was 674.81 ± 247.86 IU/L, PHOS 7.11 ± 2.27 mg/dL, and ALT 30.93 ± 9.00 IU/L, exceeding levels in healthy juveniles (Martín-Barrasa et al., 2023). Conversely, CRE in Indian Bactrian camels (1.09 ± 0.42 mg/dL) was lower than that of healthy counterparts (De et al., 2020). Through detecting blood parameters, the degree of damage and the severity of infection can be determined. Moreover, biochemical tests can provide insights into an animal’s nutrition and health status (El-Sayed et al., 2025).

When animals develop *C. perfringens* disease, host-specific disease manifestations are mainly caused by the *C. perfringens* toxins (Gulliver et al., 2023). The eight *C. perfringens* strains isolated in this study were types A and D, carrying alpha, epsilon, and enterotoxins. The alpha toxin (the most common toxin) is a zinc-containing phospholipase comprising 370 amino acids with a molecular weight of approximately 43 kDa. It comprises a membrane-bound C-domain consisting of a β-fold, a catalytic N-domain composed of an alpha helix, and a central ring domain containing a ganglioside GM1a binding site (Geier et al., 2021; Mehdizadeh Gohari et al., 2021). Alpha toxins depend on phospholipase C and sheath phospholipase activities to hydrolyze membrane phospholipids, disrupting the structural integrity of the cell membrane and resulting in cell lysis. Moreover, they influence platelet and macrophage function, inducing cytotoxicity, hemolytic activity, lethality, skin necrosis, platelet aggregation, and increased vascular permeability (Xu et al., 2024). Alpha toxins can hinder erythroid differentiation, thereby decreasing the RBC count. The mean RBC count in affected camels was higher than that in healthy camels; however, the lowest and highest values in the affected camels were significantly lower than those in healthy camels, which may be related to alpha toxins (Dos Santos et al., 2024). The ϵ-toxin is produced in the form of a weakly active prototoxin, which is subsequently removed by intestinal proteases from the N-terminal and C-terminal residues of the prototoxin, producing a mature active protein that enhances its toxin activity by 1000 times (Mehdizadeh Gohari et al., 2021). The ϵ-toxin enhances its own systemic absorption by increasing intestinal permeability. Concurrently, these toxins bind to the microvascular endothelium of tissues such as the lungs, heart, and kidneys, particularly in the presence of large amounts of ϵ-toxin in cerebral blood vessels, resulting in increased vascular permeability and plasma protein extravasation, leading to systemic edema and increased intracranial pressure, and causing acute and fatal neurological syndrome. Animals with a longer disease course showed brain softening (Finnie et al., 2020; Finnie and Uzal, 2022). Enterotoxin is a 35 kDa single peptide that lacks primary amino acid sequence homology but belongs structurally to the hemolysin β-FFT family. It consists of a C-terminal domain (cCpE) and an N-terminal domain (nCpE) (Freedman et al., 2016; Mehdizadeh Gohari et al., 2021). cCpE is a receptor-binding channel that blocks proteins, and nCpE mediates cytotoxicity and beta-pore formation. High concentrations of *cpe* can cause rapid influx of Ca^2+^, disrupt intestinal shielding function, and cause cell death (Pahle et al., 2021; Ogbu et al., 2023). When these three toxins are produced in large quantities in young camels, they are absorbed into the systemic circulation through the intestines and affect parenchymal organs (Navarro et al., 2018; Moctezuma et al., 2025). This explains the clinical signs and necropsy changes observed in young camels, including head and neck stiffness, tooth grinding, muscle tremors, leg extension, and other neurological symptoms linked to neuronal degeneration in brain tissue caused by toxins (Morris et al., 2017). Pathological changes such as edema, hemorrhage, and congestion in organs, including the heart, liver, spleen, lungs, kidneys, brain, cerebellum, pre-shoulder lymph nodes, and bladder, as well as the neuronal loss and shrinkage, are similar to those caused by *C. perfringens* in unimodal camels (Hm et al., 2024). Pathological changes, such as shedding, edema, congestion, and necrosis of intestinal villi and mucosal epithelium in the duodenum, ileum, and cecum, as well as shedding of mucosal epithelium and gastric glandular epithelium in the rumen and reticulum, were also observed, resulting in congestion. Intestinal gastritis caused by *C. perfringens* is a critical digestive disease affecting ruminants (Simpson et al., 2018). Based on clinical symptoms, necropsy results, pathological changes, and isolation and identification results (Hm et al., 2024), this study confirmed that the disease in these young camels was caused by *C. perfringens*.

Whole-genome sequencing of the eight isolated strains of *C. perfringens* revealed that the whole-genome lengths ranged from 3, 195, 592 bp to 3, 595, 833 bp, and the predicted coding sequences ranged from 2, 897 and 3, 413. The genome also included 88–96 tRNAs, including 0–10 5S, 16S, 23S RNAs, and sRNAs. Strain JLFS-2 consisted of one chromosome and three cyclic plasmids, whereas JLFS-7 consisted of one chromosome and two cyclic plasmids. Unlike Bactrian camel-derived *C. perfringens*, Zhang et al. found that deer-derived *C. perfringens* only contained 1 chromosome, 1 circular plasmid, 94 tRNAs, and 10 copies each of 5S, 16S, and 23S rRNA genes (Zhang et al., 2024).

Genomic islands are horizontally acquired transferable DNA fragments that may contain mobile elements, such as bacteriophages, integrons, and splicing transposons, which are closely associated with the pathogenicity, adaptability, and drug resistance of organisms. In this study, the JLFS-2 and JLFS-7 strains harbored 9 and 11 genomic islands, respectively, whereas each other strain predicted 3–11 gene islands (Hudson et al., 2014; Lacey et al., 2018; Zhang et al., 2024). Of the eight isolated strains, three lacked detectable CRISPR. As an acquired immune system in bacteria, the CRISPR system plays a pivotal role in defense against phage infections. The low prevalence of CRISPR may reflect a low barrier for horizontal gene transfer events or frequent interactions between phages and host cells (Nasko et al., 2019; Feng et al., 2020). The GO annotation results demonstrated that the genomes of eight strains of bacteria contained a large number of genes involved in metabolic processes, cellular processes, catalytic activities, binding, cells, cell parts, cellular anatomical entities, and protein-containing complexes, which may explain the mechanism of action of the *C. perfringens* toxin. Alpha toxin (a major virulence factor) can trigger the phospholipid metabolic pathway and influence the retraction of ileal and aortic tissues. It can also trigger the phosphatidylinositol metabolism pathway and arachidonic acid cascade reaction, thereby enhancing thromboxane production (Nagahama et al., 2019). The ϵ-toxin relies on the binding of cholesterol and sphingomyelin receptors, which exist on the outer membrane of intestinal endothelial cells and vascular cells in organs, including the brain, kidney, and liver in ruminant animals (Alves et al., 2014). The active pores formed by ϵ-toxin directly damage intestinal endothelial cells. Additionally, the side chains of aromatic amino acids of ϵ-toxin usually facilitate the binding to carbohydrate-binding proteins (Titball, 2024). *cpe* can bind to tight junction protein receptors, enhance pore formation in the plasma membrane, remove junction proteins from the cell membrane, and disrupt intercellular tight junctions (Camargo et al., 2024). The mechanisms of action of these toxins also mediate other metabolic pathways. The main metabolic pathways found in the KEGG pathway were global and overview maps, cellular community–prokaryotes, signaling molecules and interaction, translation, drug resistance, antimicrobial, and endocrine systems. Annotation against the VFDB and CARD revealed that eight isolated strains harbored seven major toxins and virulence-associated enzymes, including alpha toxin, *C. perfringens* enterotoxin, theta toxin, alpha-clostripain, kappa toxin, mu toxin, and sialidase. Among these, alpha-clostripain can influence active extracellular toxin level and is associated with muscle necrosis (Chakravorty et al., 2011; Camargo et al., 2024). Theta toxin can induce local edema, systemic shock, and multiple organ failure and is associated with hemorrhagic enteritis and intravascular hemolysis (Kiu et al., 2023). Sialidase NanJ, NanI, and NanH can enhance *C. perfringens* colonization in the intestine and trigger cytotoxic activity (Wang, 2020; Li and McClane, 2021), while mu toxin can drive tissue damage and cause gas gangrene (Camargo et al., 2024). Antibiotic resistance profiling indicated that *C. perfringens* isolates from Bactrian camels showed resistance mainly to lincomycin, clindamycin, virginiamycin M1, retapamulin, iboxamycin, vancomycin, teicoplanin, and defensin and exhibited resistance-associated patterns involving tetracycline, penicillin, and cephalosporins (Fayez et al., 2021; Ahmed et al., 2022). *C. perfringens* isolates from Bactrian camels harbored fewer antimicrobial resistance genes than isolates from other animal hosts. Research has found that ST 98, ST 41, and ST 110 were associated with human diseases, ST 54 was associated with enteritis in horses and dogs, ST 58 was associated with necrotic enteritis in poultry. Additionally, it has been confirmed that *C. perfringens* can spread among calves, dairy workers, and the environment, resulting in zoonotic diseases (Verma et al., 2020; Camargo et al., 2022). Moreover, the newly discovered ST types 1057 and 1059 have similar evolutionary relationships with human-derived strains, and Clostridium has been detected in Bactrian camel milk (Ma et al., 2025b). Whether this reflects bidirectional transmission among Bactrian camels, camel milk, and humans warrants further investigation. The predominant pathogenic ST type of Bactrian camels was mainly ST675, which clustered closely with sheep-derived strains. The newly identified ST1058 exhibited significant homology with yak- and goat-derived strains, suggesting that *C. perfringens* may spread among herbivores. Moreover, the newly discovered ST types (ST1057 and ST1059), as independent branches, indicate that *C. perfringens* of Bactrian camels has a unique genetic evolutionary path.

Although this study has provided extensive epidemiological data on the incidence of *C. perfringens* infection in Bactrian camels, these statistics are based solely on the clinical symptoms. The clinical findings are insufficient for aiding the definitive diagnosis of *C. perfringens* infection, which may have resulted in minor discrepancies between the actual and recorded incidence rates. Additionally, only a few samples were collected and isolated for this study. While the major pathogenic types of *C. perfringens* in Bactrian camels were identified, larger sample sizes and more comprehensive studies are warranted to better support disease prevention and control strategies in the Bactrian camel industry.

## 5 Conclusion

Xinjiang is a major breeding region for Bactrian camels, and the rapid development of the camel milk industry has driven many farmers and herders to increase their incomes. However, *C. perfringens* disease in young camels has not only compromised animal health but also severely affected milk production. As reflex-induced lactating animals, lactation in Bactrian camels is stimulated by suckling; therefore, the death of calves often leads to cessation of lactation within 1–2 weeks, resulting in significant economic losses.

In this study, the incidence, clinical symptoms, physiological indices, necropsy findings, pathological changes, and whole-genome sequencing of *C. perfringens* disease in Bactrian camels were systematically integrated. The incidence and mortality rates of *C. perfringens* infection in Bactrian camels were 37.89% and 54.17%, respectively. The pathogens were predominantly *C. perfringens* types A and D carrying α-toxin, ϵ-toxin, and enterotoxin. The *C. perfringens* genome in Bactrian camels exhibits distinct genomic evolutionary characteristics. These findings provide a foundation for developing vaccines and precise prevention and control strategies.

## Data Availability

The datasets presented in this study can be found in online repositories. The names of the repository/repositories and accession number(s) can be found in the article/**Supplementary Material**.

## References

[B1] Abu-Seida A. M. Hassan M. H. Abdulkarim A. Hassan E. A. (2024). Recent progress in camel research. Open Vet. J. 14, 2877–2882. doi: 10.5455/OVJ.2024.v14.i11.16. PMID: 39737032 PMC11682766

[B2] Ahmed H. A. El Bayomi R. M. Hamed R. I. Mohsen R. A. El-Gohary F. A. Hefny A. A. . (2022). Genetic relatedness, antibiotic resistance, and effect of silver nanoparticle on biofilm formation by clostridium perfringens isolated from chickens, pigeons, camels, and human consumers. Vet. Sci. 9, 109. doi: 10.3390/vetsci9030109. PMID: 35324837 PMC8949260

[B3] Al-Ruwaili M. A. Khalil O. M. Selim S. A. (2012). Viral and bacterial infections associated with camel (camelus dromedarius) calf diarrhea in north province, Saudi Arabia. Saudi J. Biol. Sci. 19, 35–41. doi: 10.1016/j.sjbs.2011.10.001. PMID: 23961160 PMC3730540

[B4] Alves G. G. MaChado de Ávila R. A. Chávez-Olórtegui C. D. Lobato F. C. F. (2014). Clostridium perfringens epsilon toxin: the third most potent bacterial toxin known. Anaerobe 30, 102–107. doi: 10.1016/j.anaerobe.2014.08.016. PMID: 25234332

[B5] Amin A. S. A. Abdoun K. A. Abdelatif A. M. (2007). Seasonal variation in blood constituents of one-humped camel (camelus dromedarius). Pak. J. Biol. Sci. PJBS 10, 1250–1256. doi: 10.3923/pjbs.2007.1250.1256. PMID: 19069924

[B6] Baker P. R. Li A. S. Griffin B. R. Gil H.-W. Orlicky D. J. Fox B. M. . (2024). Disruption in glutathione metabolism and altered energy production in the liver and kidney after ischemic acute kidney injury in mice. Sci. Rep. 14, 13862. doi: 10.1038/s41598-024-64586-4. PMID: 38879688 PMC11180093

[B7] Baums C. G. Schotte U. Amtsberg G. Goethe R. (2004). Diagnostic multiplex PCR for toxin genotyping of clostridium perfringens isolates. Vet. Microbiol. 100, 11–16. doi: 10.1016/S0378-1135(03)00126-3. PMID: 15135508

[B8] Camargo A. Guerrero-Araya E. Castañeda S. Vega L. Cardenas-Alvarez M. X. Rodríguez C. . (2022). Intra-species diversity of clostridium perfringens: a diverse genetic repertoire reveals its pathogenic potential. Front. Microbiol. 13, 952081. doi: 10.3389/fmicb.2022.952081. PMID: 35935202 PMC9354469

[B9] Camargo A. Ramírez J. D. Kiu R. Hall L. J. Muñoz M. (2024). Unveiling the pathogenic mechanisms of clostridium perfringens toxins and virulence factors. Emerging Microbes Infect. 13, 2341968. doi: 10.1080/22221751.2024.2341968. PMID: 38590276 PMC11057404

[B10] Chakravorty A. Awad M. M. Hiscox T. J. Cheung J. K. Carter G. P. Choo J. M. . (2011). The cysteine protease α-clostripain is not essential for the pathogenesis of clostridium perfringens-mediated myonecrosis. PloS One 6, e22762. doi: 10.1371/journal.pone.0022762. PMID: 21829506 PMC3146509

[B11] De U. K. Chander V. Akhilesh N. Mahajan S. Sharma G. K. Nandi S. . (2020). Alterations of hemogram, serum biochemistry, oxidative/nitrosative balance, and copper/zinc homeostasis in dromedary camels naturally infected with poxvirus. Trop. Anim. Health Prod. 52, 2997–3003. doi: 10.1007/s11250-020-02318-2. PMID: 32519073

[B12] Dos Santos B. A. Barbosa B. E. P. Alves A. C. T. Miranda B. P. Santos G. F. Balthazar D. A. (2024). Clostridium perfringens alpha toxin enteritis associated with pulmonary disease in a neotropical otter (lontra longicaudis, olfers 1818) under human care. Braz. J. Vet. Med. 46, e006724. doi: 10.29374/2527-2179.bjvm006724. PMID: 39748914 PMC11694838

[B13] Elhelw H. A. El Fadeel M. R. A. El-Sergany E. Allam A. Elbayoumy M. K. El-Kattan A. M. . (2022). Preparation and field study of combined vaccine against clostridium perfringens type a and bovine viral diarrhea virus in camels. Clin. Exp. Vaccine Res. 11, 30–42. doi: 10.7774/cevr.2022.11.1.30. PMID: 35223663 PMC8844669

[B14] Elnar A. G. Kim G.-B. (2021). Complete genome sequence of clostridium perfringens B20, a bacteriocin-producing pathogen. J. Anim. Sci. Technol. 63, 1468–1472. doi: 10.5187/jast.2021.e113. PMID: 34957460 PMC8672250

[B15] El-Sayed A. A.-M. El-Sayed A. A. Ali M. E. Eissa A. Askar A. R. Mousa S. (2025). Pulsed-wave doppler echocardiographic and hematobiochemical profiles of clinically healthy racing dromedary camels. Open Vet. J. 15, 994–1008. doi: 10.5455/OVJ.2025.v15.i2.48. PMID: 40201822 PMC11974305

[B16] Fayez M. El-Ghareeb W. R. Elmoslemany A. Alsunaini S. J. Alkafafy M. Alzahrani O. M. . (2021). Genotyping and antimicrobial susceptibility of clostridium perfringens and clostridioides difficile in camel minced meat. Pathog. (Basel Switz.) 10, 1640. doi: 10.3390/pathogens10121640. PMID: 34959595 PMC8708398

[B17] Fayez M. Elsohaby I. Al-Marri T. Zidan K. Aldoweriej A. El-Sergany E. . (2020). Genotyping and antimicrobial susceptibility of clostridium perfringens isolated from dromedary camels, pastures and herders. Comp. Immunol. Microbiol. Infect. Dis. 70, 101460. doi: 10.1016/j.cimid.2020.101460. PMID: 32145560

[B18] Fayez M. Suleiman M. B. Al M. A. (2013). Clostridium perfringens enterotoxaemia In camel (camelusdromedarius) calves. Int. J. Adv. Res. 1, 239–247.

[B19] Feng Y. Fan X. Zhu L. Yang X. Liu Y. Gao S. . (2020). Phylogenetic and genomic analysis reveals high genomic openness and genetic diversity of clostridium perfringens. Microb. Genomics 6, mgen000441. doi: 10.1099/mgen.0.000441. PMID: 32975504 PMC7660258

[B20] Feng H. Wu K. Yuan Y. Fang M. Wang J. Li R. . (2024). Genomic analysis of clostridium perfringens type D isolates from goat farms. Vet. Microbiol. 294, 110105. doi: 10.1016/j.vetmic.2024.110105. PMID: 38729094

[B21] Finnie J. W. Navarro M. A. Uzal F. A. (2020). Pathogenesis and diagnostic features of brain and ophthalmic damage produced by clostridium perfringens type D epsilon toxin. J. Vet. Diagn. Investig. Off. Publ. Am. Assoc. Vet. Lab. Diagn. Inc 32, 282–286. doi: 10.1177/1040638719900190. PMID: 31955669 PMC7081509

[B22] Finnie J. W. Uzal F. A. (2022). Pathology and pathogenesis of brain lesions produced by clostridium perfringens type D epsilon toxin. Int. J. Mol. Sci. 23, 9050. doi: 10.3390/ijms23169050. PMID: 36012315 PMC9409160

[B23] Freedman J. C. Shrestha A. McClane B. A. (2016). Clostridium perfringens enterotoxin: action, genetics, and translational applications. Toxins 8, 73. doi: 10.3390/toxins8030073. PMID: 26999202 PMC4810218

[B24] Geier R. R. Rehberger T. G. Smith A. H. (2021). Comparative genomics of clostridium perfringens reveals patterns of host-associated phylogenetic clades and virulence factors. Front. Microbiol. 12, 649953. doi: 10.3389/fmicb.2021.649953. PMID: 34177831 PMC8220089

[B25] Grenda T. Jarosz A. Sapała M. Grenda A. Patyra E. Kwiatek K. (2023). Clostridium perfringens—opportunistic foodborne pathogen, its diversity and epidemiological significance. Pathogens 12, 768. doi: 10.3390/pathogens12060768. PMID: 37375458 PMC10304509

[B26] Gulliver E. L. Adams V. Marcelino V. R. Gould J. Rutten E. L. Powell D. R. . (2023). Extensive genome analysis identifies novel plasmid families in clostridium perfringens. Microb. Genomics 9, mgen000995. doi: 10.1099/mgen.0.000995. PMID: 37079454 PMC10210947

[B27] Gupta P. Asopa S. Dadhich H. Pankaj D. K. (2015). Types, pattern and morphology of enteritis prevalent in pigs (sus scrofa domesticus) of rajasthan. ournal Camel Pract. Researc SP-11, 4005–4008. doi: 10.5958/2277-8934.2015.00048.X

[B28] Heidari F. Sharifiyazdi H. Nazifi S. Ghane M. Hosseinzadeh S. (2023). Coxiella burnetii and borrelia spp. in peripheral blood of dromedary camels in fars, Iran: molecular characterization, hematological parameters, and acute-phase protein alterations. Iran. J. Vet. Res. 24, 174–181. doi: 10.22099/IJVR.2023.46933.6746. PMID: 38269010 PMC10804426

[B29] Heidarpanah S. Li K. Thibodeau A. Meniaï I. Parreira V. R. Quessy S. . (2024). Genomic diversity and virulence factors of clostridium perfringens isolated from healthy and necrotic enteritis-affected broiler chicken farms in quebec province. Microorganisms 12, 2624. doi: 10.3390/microorganisms12122624. PMID: 39770825 PMC11677781

[B30] Hm A. S H. Mz A. Ab S. S A. M M. . (2024). Molecular identification of different toxinogenic strains of clostridium perfringens and histo-pathological observations of camels died of per-acute entero-toxemia. Heliyon 10, 1–9. doi: 10.1016/j.heliyon.2024.e27859. PMID: 38533056 PMC10963320

[B31] Hudson C. M. Bent Z. W. Meagher R. J. Williams K. P. (2014). Resistance determinants and mobile genetic elements of an NDM-1-encoding klebsiella pneumoniae strain. PloS One 9, e99209. doi: 10.1371/journal.pone.0099209. PMID: 24905728 PMC4048246

[B32] Hussain R. Guangbin Z. Abbas R. Z. Siddique A. B. Mohiuddin M. Khan I. . (2022). Clostridium perfringens types a and D involved in peracute deaths in goats kept in cholistan ecosystem during winter season. Front. Vet. Sci. 9, 849856. doi: 10.3389/fvets.2022.849856. PMID: 35372540 PMC8971777

[B33] Jiang Z. Su W. Yang M. Li W. Gong T. Zhang Y. . (2022). Screening of bacteria inhibiting clostridium perfringens and assessment of their beneficial effects *In vitro* and *In vivo* with whole genome sequencing analysis. Microorganisms 10, 2056. doi: 10.3390/microorganisms10102056. PMID: 36296333 PMC9609858

[B34] Kiu R. Hall L. J. (2018). An update on the human and animal enteric pathogen clostridium perfringens. Emerging Microbes Infect. 7, 141. doi: 10.1038/s41426-018-0144-8. PMID: 30082713 PMC6079034

[B35] Kiu R. Shaw A. G. Sim K. Acuna-Gonzalez A. Price C. A. Bedwell H. . (2023). Particular genomic and virulence traits associated with preterm infant-derived toxigenic clostridium perfringens strains. Nat. Microbiol. 8, 1160–1175. doi: 10.1038/s41564-023-01385-z. PMID: 37231089 PMC10234813

[B36] Kong N. Chen G. Wang H. Li J. Yin S. Cao X. . (2021). Blood leukocyte count as a systemic inflammatory biomarker associated with a more rapid spirometric decline in a large cohort of iron and steel industry workers. Respir. Res. 22, 254. doi: 10.1186/s12931-021-01849-y. PMID: 34565362 PMC8467242

[B37] Lacey J. A. Allnutt T. R. Vezina B. Van T. T. H. Stent T. Han X. . (2018). Whole genome analysis reveals the diversity and evolutionary relationships between necrotic enteritis-causing strains of clostridium perfringens. BMC Genomics 19, 379. doi: 10.1186/s12864-018-4771-1. PMID: 29788909 PMC5964661

[B38] Li J. McClane B. A. (2021). NanH is produced by sporulating cultures of clostridium perfringens type F food poisoning strains and enhances the cytotoxicity of C. perfringens enterotoxin. Msphere 6, e00176-21. doi: 10.1128/mSphere.00176-21. PMID: 33910991 PMC8092135

[B39] Li J. Paredes-Sabja D. Sarker M. R. McClane B. A. (2016). Clostridium perfringens sporulation and sporulation-associated toxin production. Microbiol. Spectr. 4, 331–347. doi: 10.1128/microbiolspec.TBS-0022-2015. PMID: 27337447 PMC4920134

[B40] Ma W. Yao H. Zhang L. Zhang Y. Wang Y. Wang W. . (2025a). Transcriptomics-based study of immune genes associated with subclinical mastitis in bactrian camels. Vet. Sci. 12, 121. doi: 10.3390/vetsci12020121. PMID: 40005880 PMC11861070

[B41] Ma W. Zhang L. Yao H. Zhang Y. Wang W. Liu Y. . (2025b). A study on the changing law of bacterial communities in the milk of bactrian camels with subclinical mastitis. Microorganisms 13, 1394. doi: 10.3390/microorganisms13061394. PMID: 40572282 PMC12196136

[B42] Mahmood F. Khan A. Hussain R. Khan I. A. Abbas R. Z. Ali H. M. . (2017). Patho-bacteriological investigation of an outbreak of mycoplasma bovis infection in calves - emerging stealth assault. Microb. Pathogen. 107, 404–408. doi: 10.1016/j.micpath.2017.04.003. PMID: 28389347

[B43] Martín-Barrasa J. L. Tejedor-Junco M. T. Cabrera S. Morales M. Melián A. Corbera J. A. (2023). Hematological and biochemical blood reference values for canary island camels (camelus dromedarius), an endangered dromedary species. Saudi J. Biol. Sci. 30, 103677. doi: 10.1016/j.sjbs.2023.103677. PMID: 37213697 PMC10196958

[B44] Mehdizadeh Gohari I. A Navarro M. Li J. Shrestha A. Uzal F. A McClane B. (2021). Pathogenicity and virulence of clostridium perfringens. Virulence 12, 723–753. doi: 10.1080/21505594.2021.1886777. PMID: 33843463 PMC8043184

[B45] Moctezuma K. Acevedo H. D. Henderson E. E. Asin J. Adaska J. M. Uzal F. A. (2025). Enterotoxemia in a 2-day-old lamb produced by a clostridium perfringens type D lambda toxin–positive strain. J. Vet. Diagn. Invest. 37 (3), 504–506. doi: 10.1177/10406387251320943. PMID: 40017064 PMC11869215

[B46] Moebuu N. Aiuurzana N. Dashdava N. Ipatenko N. G. (1966). infectious enterotoxemia of camels caused by C. perfringens, type C. Veterinariia 43, 32–35. 4299662

[B47] Mohammadpour R. Champour M. Tuteja F. Mostafavi E. (2020). Zoonotic implications of camel diseases in Iran. Vet. Med. Sci. 6, 359–381. doi: 10.1002/vms3.239. PMID: 32160657 PMC7397890

[B48] Morris W. E. Goldstein J. Redondo L. M. Cangelosi A. Geoghegan P. Brocco M. . (2017). Clostridium perfringens epsilon toxin induces permanent neuronal degeneration and behavioral changes. Toxicon: Off. J. Int. Soc Toxinology 130, 19–28. doi: 10.1016/j.toxicon.2017.02.019. PMID: 28237716

[B49] Moustafa S. Zakaria I. Moustafa A. AboSakaya R. Selim A. (2022). Bacteriological and serological investigation of clostridium perfringens in lambs. Sci. Rep. 12, 19715. doi: 10.1038/s41598-022-21918-6. PMID: 36385107 PMC9669049

[B50] Mubarak A. G. Khalifa F. A. Elsobky Y. Abdel-Rady A. Felefel W. Saad A. H. . (2024). Sudden death due to enterotoxemia among arabian camels (camelus dromedaries) and associated risk factors. Open Vet. J. 14, 1942–1951. doi: 10.5455/OVJ.2024.v14.i8.23. PMID: 39308733 PMC11415913

[B51] Nagahama M. Takehara M. Rood J. I. (2019). Histotoxic clostridial infections. Microbiol. Spectr. 7, 1–17. doi: 10.1128/microbiolspec.GPP3-0024-2018. PMID: 31350831 PMC10957196

[B52] Nasko D. J. Ferrell B. D. Moore R. M. Bhavsar J. D. Polson S. W. Wommack K. E. (2019). CRISPR spacers indicate preferential matching of specific virioplankton genes. Mbio 10, e02651-18. doi: 10.1128/mBio.02651-18. PMID: 30837341 PMC6401485

[B53] Navarro M. A. Li J. Beingesser J. McClane B. A. Uzal F. A. (2020). The agr-like quorum-sensing system is important for clostridium perfringens type a strain ATCC 3624 to cause gas gangrene in a mouse model. mSphere 5, e00500-20. doi: 10.1128/mSphere.00500-20. PMID: 32554714 PMC7300355

[B54] Navarro M. A. McClane B. A. Uzal F. A. (2018). Mechanisms of action and cell death associated with clostridium perfringens toxins. Toxins 10, 212. doi: 10.3390/toxins10050212. PMID: 29786671 PMC5983268

[B55] Ogbu C. P. Kapoor S. Vecchio A. J. (2023). Structural basis of clostridium perfringens enterotoxin activation and oligomerization by trypsin. Toxins 15, 637. doi: 10.3390/toxins15110637. PMID: 37999500 PMC10674488

[B56] Omer S. A. Al-Olayan E. M. Babiker S. E. H. Aljulaifi M. Z. Alagaili A. N. Mohammed O. B. (2020). Genotyping of clostridium perfringens isolates from domestic livestock in Saudi Arabia. BioMed. Res. Int. 2020, 9035341. doi: 10.1155/2020/9035341. PMID: 32280706 PMC7128066

[B57] Pahle J. Kobelt D. Aumann J. Behrens D. Daberkow O. Mokritzkij M. . (2021). Effective oncoleaking treatment of pancreatic cancer by claudin-targeted suicide gene therapy with clostridium perfringens enterotoxin (CPE). Cancers 13, 4393. doi: 10.3390/cancers13174393. PMID: 34503203 PMC8431234

[B58] Quinton L. J. Walkey A. J. Mizgerd J. P. (2018). Integrative physiology of pneumonia. Physiol. Rev. 98, 1417–1464. doi: 10.1152/physrev.00032.2017. PMID: 29767563 PMC6088146

[B59] Santos R. A. N. D. Abdel-Nour J. McAuley C. Moore S. C. Fegan N. Fox E. M. (2022). Clostridium perfringens associated with dairy farm systems show diverse genotypes. Int. J. Food Microbiol. 382, 109933. doi: 10.1016/j.ijfoodmicro.2022.109933. PMID: 36166891

[B60] Shimada K. Mitchison T. J. (2019). Unsupervised identification of disease states from high‐dimensional physiological and histopathological profiles. Mol. Syst. Biol. 15, e8636. doi: 10.15252/msb.20188636. PMID: 30782979 PMC6380462

[B61] Simpson K. M. Callan R. J. Van Metre D. C. (2018). Clostridial abomasitis and enteritis in ruminants. Vet. Clin. North. Am. Food. Anim. Pract. 34, 155–184. doi: 10.1016/j.cvfa.2017.10.010. PMID: 29421028 PMC7127689

[B62] Tao W. Aniwar L. ZuliPicar A. Tulafu H. Zhang R. Liu B. . (2023). Analysis of genetic diversity and population structure of tarim and junggar bactrian camels based on simplified GBS genome sequencing. Anim. Open Access J. MDPI 13, 2349. doi: 10.3390/ani13142349. PMID: 37508126 PMC10376019

[B63] Titball R. W. (2024). The molecular architecture and mode of action of clostridium perfringens ϵ-toxin. Toxins 16, 180. doi: 10.3390/toxins16040180. PMID: 38668605 PMC11053738

[B64] Verma A. K. Abdel-Glil M. Y. Madesh A. Gupta S. Karunakaran A. C. Inbaraj S. . (2020). Multilocus sequence typing of clostridium perfringens strains from neonatal calves, dairy workers and associated environment in India. Anaerobe 63, 102212. doi: 10.1016/j.anaerobe.2020.102212. PMID: 32413405

[B65] Wahdan A. Elhaig M. M. (2024). Epidemiology and diagnostic accuracy of clostridium perfringens toxins in the intestinal contents of camels, sheep, and cattle: a cross-sectional study in dakahlia governorate, Egypt. Trop. Anim. Health Prod. 56, 205. doi: 10.1007/s11250-024-04034-7. PMID: 39001933 PMC11246295

[B66] Wang Y. (2020). Sialidases from clostridium perfringens and their inhibitors. Front. Cell. Infect. Microbiol. 9, 462. doi: 10.3389/fcimb.2019.00462. PMID: 31998664 PMC6966327

[B67] Wernery U. Ali M. Wernery R. Seifert H. S. (1992). Severe heart muscle degeneration caused by clostridium perfringens type a in camel calves (camelus dromedarius). Rev. Elev. Med. Vet. Pays Trop. 45, 255–259. 1339990

[B68] Wu K. Feng H. Ma J. Wang B. Feng J. Zhang H. . (2022). Prevalence, toxin-typing and antimicrobial susceptibility of clostridium perfringens in sheep with different feeding modes from gansu and qinghai provinces, China. Anaerobe 73, 102516. doi: 10.1016/j.anaerobe.2022.102516. PMID: 35026419

[B69] Xu C. She Y. Fu F. Xu C. Peng K. (2024). Review of advances in molecular structure and biological function of alpha toxin of clostridium perfringens. Can. J. Vet. Res. = Rev. Can. Rech. Vet. 88, 138–144. PMC11418754 39355682

[B70] Yan Z. Wang H. Zhu Y. Wang X. Wu Y. Wang Y. . (2025). Molecular epidemiology of type F clostridium perfringens among diarrheal patients and virulence-resistance dynamics - 11 provinces, China. China CDC wkly. 7, 69–76. doi: 10.46234/ccdcw2025.013. PMID: 39867821 PMC11757904

[B71] Yao H. Dou Z. Zhao Z. Liang X. Yue H. Ma W. . (2023). Transcriptome analysis of the bactrian camel (camelus bactrianus) reveals candidate genes affecting milk production traits. BMC Genomics 24, 660. doi: 10.1186/s12864-023-09703-9. PMID: 37919661 PMC10621195

[B72] Younan M. Gluecks I. V. (2007). Clostridium perfringens type b enterotoxaemia in a Kenyan camel. J. Camel Pract. Res. 14, 65–67.

[B73] Zafar Khan M. U. Khalid S. Humza M. Yang S. Alvi M. A. Munir T. . (2022). Infection dynamics of clostridium perfringens fingerprinting in buffalo and cattle of punjab province, Pakistan. Front. Vet. Sci. 9, 762449. doi: 10.3389/fvets.2022.762449. PMID: 35937290 PMC9353052

[B74] Zeng X. Liu B. Zhou J. Dai Y. Han C. Wang L. . (2021). Complete genomic sequence and analysis of β2 toxin gene mapping of clostridium perfringens JXJA17 isolated from piglets in China. Sci. Rep. 11, 475. doi: 10.1038/s41598-020-79333-8. PMID: 33436645 PMC7804025

[B75] Zhang Z. Wang X. Li S. Fu Y. Li Y. Nawaz S. . (2024a). Isolation of a virulent clostridium perfringens strain from elaphurus davidianus and characterization by whole-genome sequence analysis. Curr. Issues Mol. Biol. 46, 7169–7186. doi: 10.3390/cimb46070427. PMID: 39057068 PMC11276296

[B76] Zhu X. Huang Y. Shi Y. Gao X. Chen D. Liu C. . (2025). Comparative genomic analysis of food-animal-derived and human-derived clostridium perfringens isolates from markets in shandong, China. Front. Microbiol. 16, 1543511. doi: 10.3389/fmicb.2025.1543511. PMID: 40236475 PMC11996926

