# Supplementary figures and images for: Type A and D Clostridium perfringens with unique genetic evolution characteristics cause mortality in juvenile Bactrian camels
Source: Front Cell Infect Microbiol. 2026 May 29;16:1787432. doi: 10.3389/fcimb.2026.1787432 (PMC13259845; doi:10.3389/fcimb.2026.1787432)

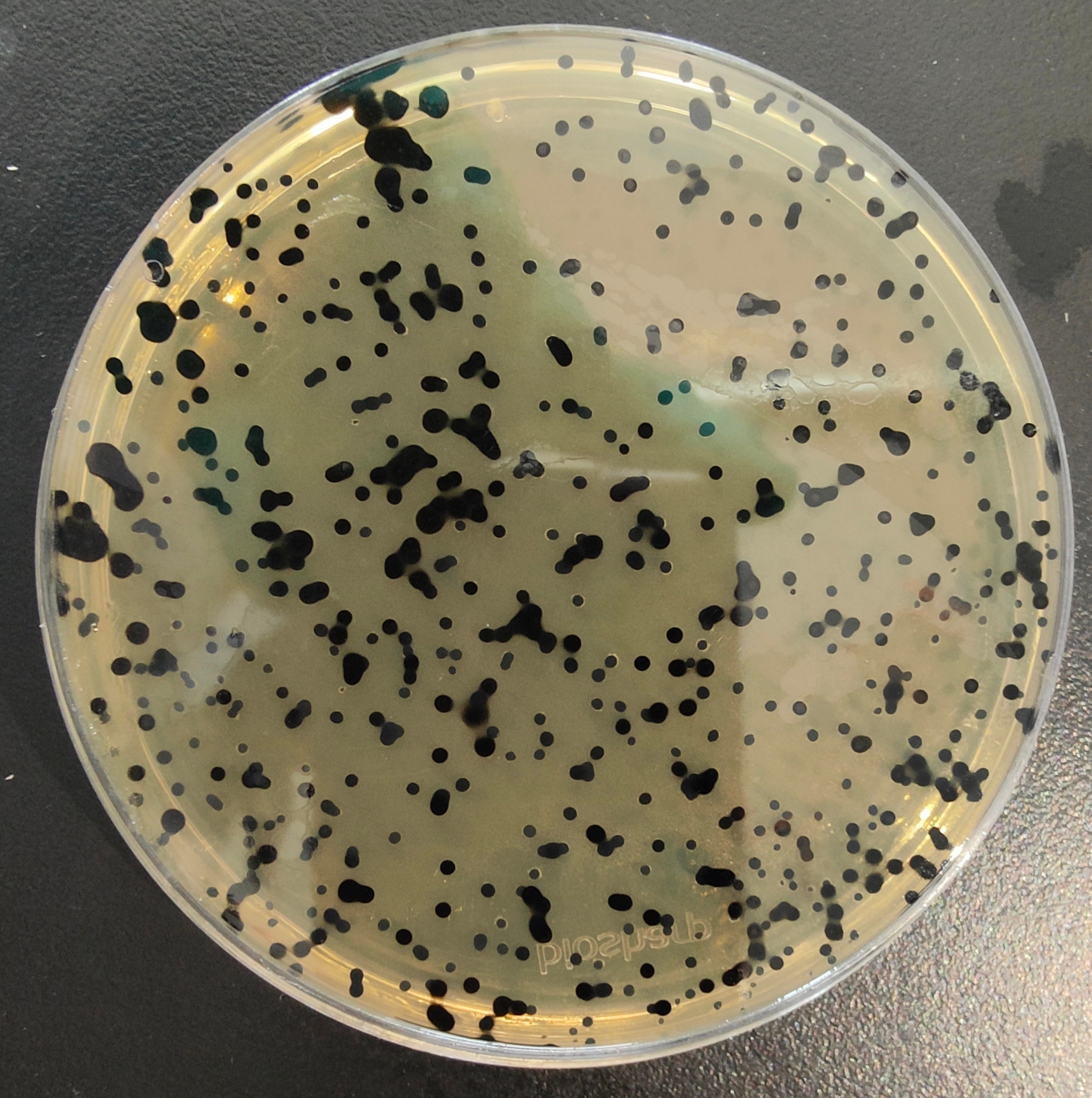

Supplement: Supplementary file 6 [file Image1.jpeg]

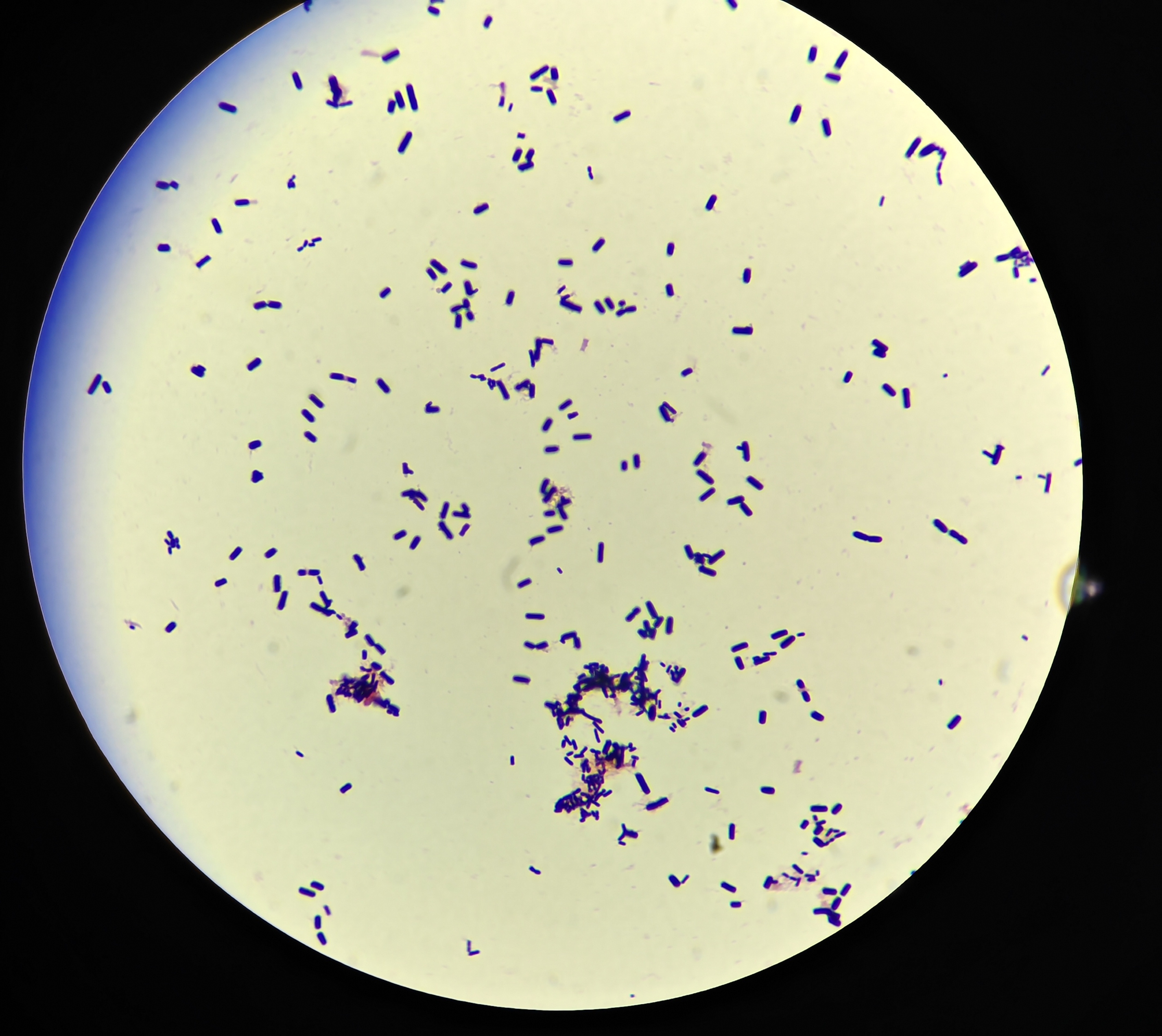

Supplement: Supplementary file 7 [file Image2.jpeg]

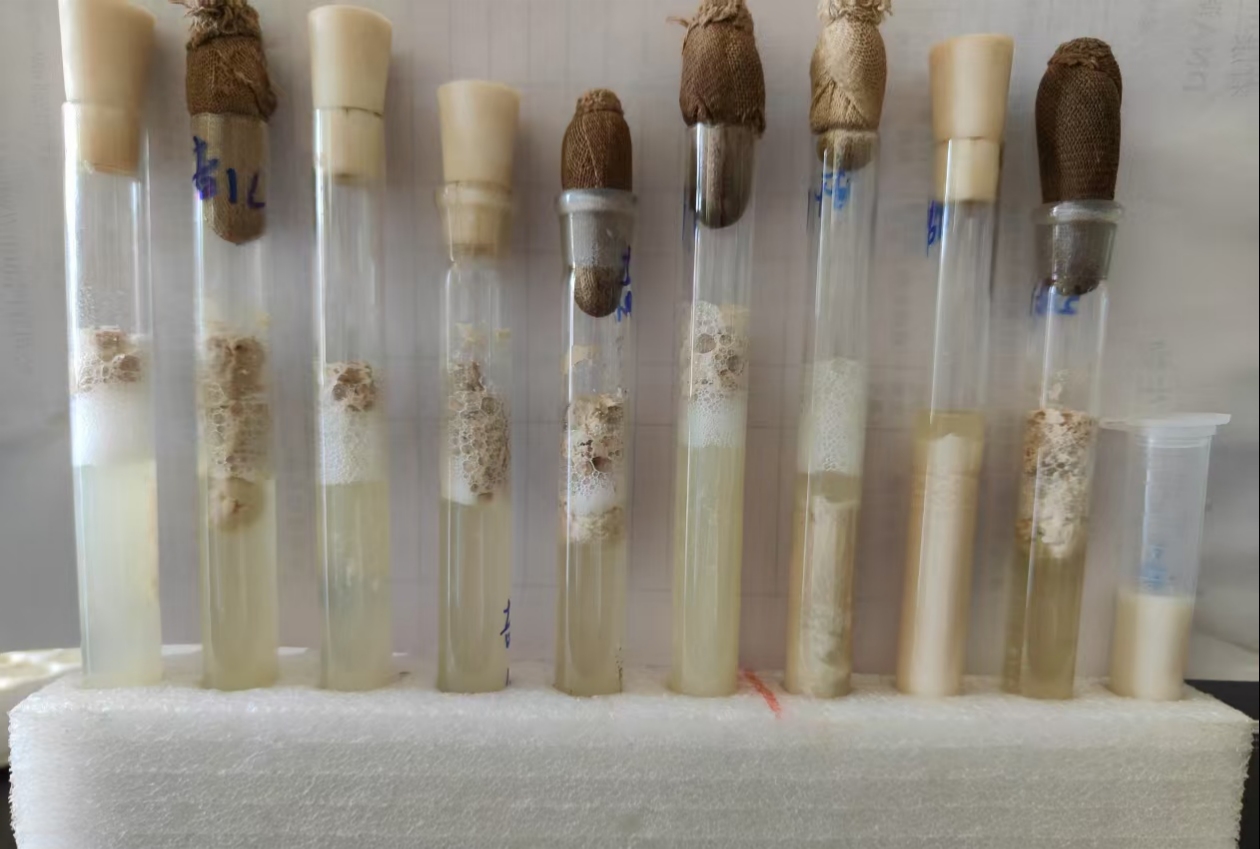

Supplement: Supplementary file 8 [file Image3.jpeg]

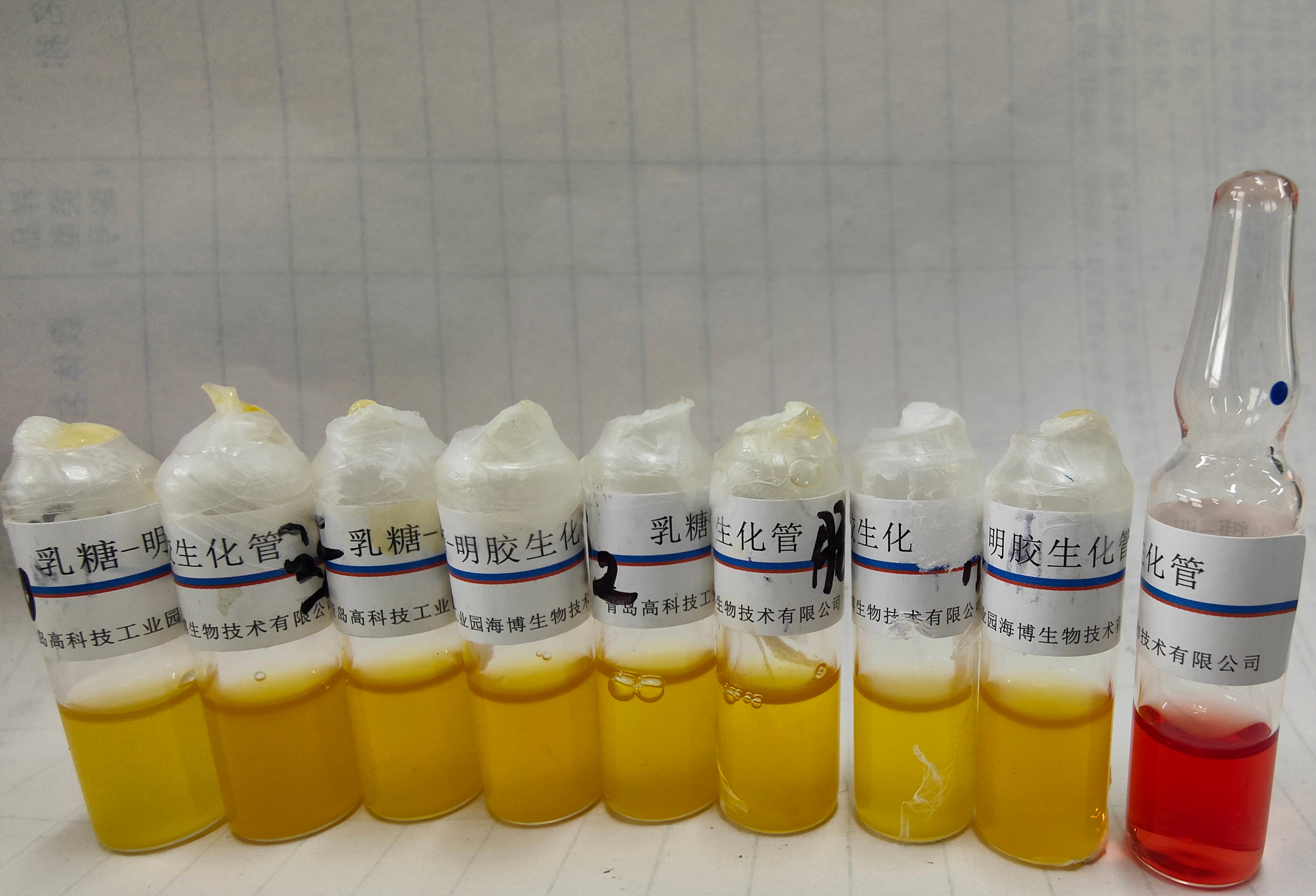

Supplement: Supplementary file 9 [file Image4.jpeg]

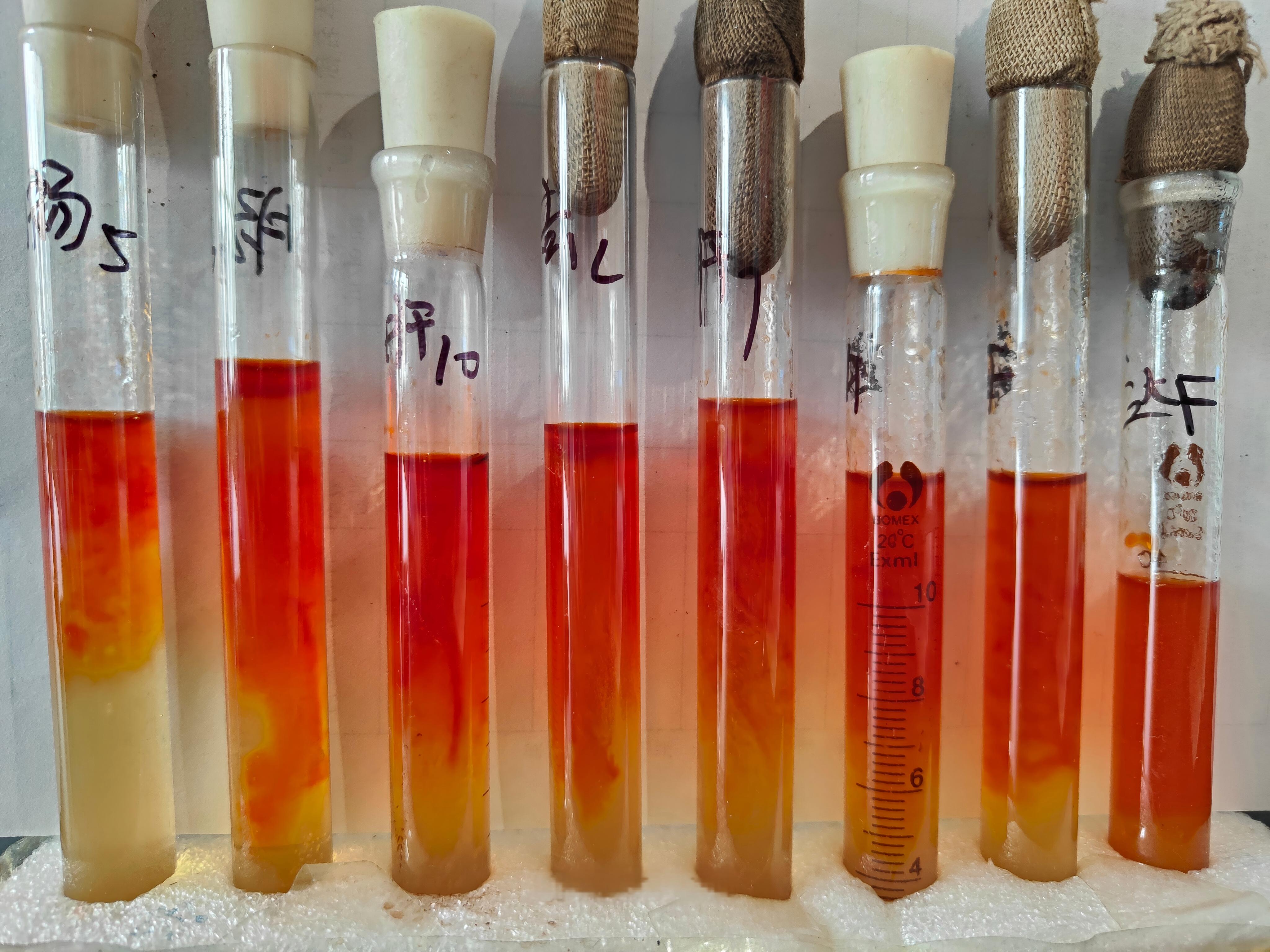

Supplement: Supplementary file 10 [file Image5.jpeg]
